# Endovascular Therapy, Open Surgical Bypass, and Conduit Types for Index Treatment of Claudication

**DOI:** 10.1001/jamanetworkopen.2025.33352

**Published:** 2025-10-16

**Authors:** Tiffany R. Bellomo, Gabriel Jabbour, Mohit Manchella, Srihari K. Lella, Shravan Animilli, Yuanyuan Zhao, Jiwoo Lee, C. Y. Maximilian Png, Bianca Mulaney, Brandon Gaston, Falen Demsas, Micah Thornton, Matthew J. Eagleton, Sunita D. Srivastava, Anahita Dua, Nikolaos Zacharias

**Affiliations:** 1 Division of Vascular and Endovascular Surgery, Department of Surgery, Massachusetts General Hospital, Boston; 2 Harvard Medical School, Massachusetts General Hospital, Boston; 3 Georgetown University School of Medicine, Washington, DC; 4 Society for Vascular Surgery, Rosemont, Illinois

## Abstract

**Importance:**

Despite advances in infrainguinal revascularization and established guidelines, direct comparisons between endovascular and open surgical strategies remain limited due to challenges in trial enrollment, anatomical variability, and lack of long-term follow-up.

**Objective:**

To compare the outcomes of index endovascular procedures vs open surgical bypass among patients with peripheral arterial disease with claudication.

**Design, Setting, and Participants:**

This retrospective cohort study, spanning from January 2007 to October 2024, enrolled participants with femoropopliteal peripheral arterial disease who underwent nonemergent index interventions for the indication of claudication into the multicenter national registry Vascular Quality Initiative.

**Exposures:**

Category of revascularization procedure and type of conduit.

**Main Outcomes and Measures:**

The primary outcome was major amputation above the ankle at 1 year.

**Results:**

In total, 22 328 patients were included (mean [SD] age, 67.7 [10.3] years for the entire cohort; 64.4 [10.0] years for 4474 patients in the open surgical bypass group, and 68.6 [10.2] years for 17 854 patients in the endovascular procedures group). Patients had a median (IQR) follow-up duration of 1.0 (0.9-1.4) years (maximum, 8.3 years). Most patients were male (open 3334 [74.5%] vs endovascular 10 938 [61.3%]; *P* < .001), and both groups had similar obesity rates (1589 [35.6%] for open vs 6284 [35.2%] for endovascular; *P* = .14). Cardiovascular comorbidities were prevalent in both groups (eg, hypertension, 3693 [82.5%] for open and 15 750 [88.2%] for endovascular). Endovascular interventions were associated with a significantly lower major amputation risk at 1 year (hazard ratio [HR], 0.67 [95% CI, 0.48-0.96]; *P* = .03) but also associated with higher risk of death at 1 year (HR, 2.09 [95% CI, 1.97-2.23]; *P* < .001) that was unchanged after propensity matching. Among open bypass procedures, prosthetic conduits were not associated with higher major amputation risk (HR, 1.66 [95% CI, 0.91-3.03]; *P* = .10) and had equivalent patency (HR, 0.96 [95% CI, 0.90-1.02]; *P* = .23) at 1 year compared with great saphenous vein (GSV) conduits. Among single segment GSV conduits, reversed GSV conduits were associated with the lowest major amputation risk (HR, 0.40 [95% CI, 0.20-0.83]; *P* = .01) at 1 year compared with in situ and transposed GSV conduits (likelihood ratio test, *P* = .002). Among prosthetic conduits, Dacron grafts were associated with higher major amputation risk than polytetrafluoroethylene grafts (HR, 4.78 [95% CI, 1.02-22.30] vs HR, 1.59 [95% CI, 1.15-2.20]; likelihood ratio test, *P* = .002).

**Conclusions and Relevance:**

In this cohort study of 22 328 participants who underwent femoropopliteal index interventions for claudication, endovascular procedures were associated with lower rates of major amputation at 1 year compared with open surgical bypass approaches. The findings suggest that if an open approach is needed, reversed GSV conduits should be prioritized in surgical bypass.

## Key Points

**Question:**

Are endovascular interventions associated with a higher major amputation risk than open surgical bypass among patients with peripheral arterial disease with claudication?

**Findings:**

This cohort study involving 22 328 patients showed that endovascular procedures were associated with a lower rate of major amputation at 1 year compared with open surgical bypass. Among open bypass approaches, reversed great saphenous vein (GSV) conduits were associated with the lowest risk of major amputation.

**Meaning:**

These findings suggest that patients with claudication may benefit most from endovascular-first intervention and subsequent open bypass using reversed GSV conduits.

## Teaser

This cohort study compares the outcomes of index endovascular therapy with open surgical bypass procedures and conduit type among patients with peripheral arterial disease with claudication.

## Introduction

Peripheral arterial disease (PAD) is a substantial global health concern, affecting an estimated 8 to 10 million people in the US alone.^1^ Symptomatic PAD often manifests as claudication, characterized by leg pain during activity that resolves with rest. If left untreated, PAD can progress to chronic limb-threatening ischemia (CLTI), a more severe condition marked by rest pain or the development of foot wounds, which can lead to limb loss if not addressed promptly.^2,3^ While much of the current PAD research has focused on patients with acute limb ischemia and CLTI, approximately 1 in 5 patients with PAD have claudication that may require revascularization.^4^ The Society for Vascular Surgery (SVS) guidelines recommend revascularization for patients with lifestyle-limiting claudication whose condition has not responded adequately to medical management and exercise therapy.^5^

The landscape of infrainguinal revascularization for patients with claudication has evolved substantially over the past few decades, with endovascular interventions gaining prominence alongside traditional open surgical approaches.^6^ This shift has been driven by technological advancements and the potential for reduced perioperative morbidity associated with endovascular procedures, which is particularly appealing for patients with claudication, who often seek less invasive treatment options.^4,7,8^ There is ongoing debate regarding the durability of these endovascular interventions, as they may ultimately lead to the need for an open bypass, which has the potential to provide lifelong durability for the patient. Comparison of open and endovascular approaches for lower extremity revascularization in patients with intermittent claudication has been controversial and limited by sample size.^6,9,10^ Comparisons of conduit type have also been limited, and there is little evidence to support one particular vein configuration over another.

Despite the technological advances of infrainguinal revascularization and the wealth of guidelines available, a direct comparison of endovascular and open strategies remains limited primarily due to enrollment challenges in comparative trials, variations in vascular anatomy, and absence of long-term follow-up. The Vascular Quality Initiative (VQI) is a large comprehensive international registry that offers the opportunity to study patients who specifically underwent infrainguinal interventions with follow-up often beyond 1 year. In this study, we compared index endovascular procedures and open surgical bypass, including conduit types, among patients with claudication to assess the risk of major amputation.

## Methods

### Study Population

This retrospective cohort study used the SVS VQI infrainguinal and peripheral vascular intervention (PVI) registries^11^ and followed Strengthening the Reporting of Observational Studies in Epidemiology (STROBE) reporting guidelines. The study period was defined as the inception of the database in January 2007 to October 2024. Analyses were performed under a national research advisory committee proposal. The Mass General Brigham Human Research Committee Institutional Review Board approved this study and waived the need for informed consent because the data are deidentified and publicly available on reasonable request from VQI.

The VQI is an international registry that includes a network of vascular specialists founded to improve the quality and safety of vascular care by sharing procedure data. The inclusion criteria for both infrainguinal and PVI datasets were participants over 18 years of age, with procedures performed in the US, claudication as indication for procedure, first infrainguinal revascularization procedure performed, and nonemergent and nonurgent procedures (eFigure 1 in Supplement 1). Exclusion criteria for both infrainguinal and PVI datasets were procedures performed internationally, procedure indications other than claudication, history of amputation, concurrent endarterectomies, procedure status of emergent or urgent, and absent comorbidity data to ensure a complete case analysis (eTable 1 in Supplement 1). Further details on inclusion and exclusion criteria, as well as the VQI-based definition of claudication, are provided in the eMethods in Supplement 1.

### Exposure Variables

The primary exposure of index procedure type was a binary variable classified as open surgical bypass procedure or endovascular intervention (eTable 2 in Supplement 2). The subsequent set of analyses were conduit within the infrainguinal or open surgical bypass dataset only. Conduits were divided into binary variables of prosthetic (polytetrafluoroethylene [PTFE] or Dacron) and great saphenous vein (GSV) conduits (reversed, in situ, and transposed), with analysis limited to these types due to low case numbers for other vein or spliced conduits.

### Outcome Definitions

All outcomes were obtained from the VQI Long-Term Follow-Up datasets, which capture follow-up data for up to 1 year following the index procedure (eTable 3 in Supplement 2). Our primary outcome was major amputation above the ankle, including below the knee amputation or above the knee amputation, ipsilateral to the limb that underwent the index intervention at 1 year after the index procedure. All participants without amputation outcome data were excluded, which consisted of 7 of 4848 patients who underwent the open intervention and 12 308 of 30 368 patients who underwent the endovascular intervention. Secondary outcomes included myocardial infarction, death, and readmission in both datasets and primary patency and return to the operating room specific to the infrainguinal dataset, as outlined in the eMethods in Supplement 1. All analyzed outcomes had less than 1% missing data, minimizing the potential for bias due to incomplete outcome ascertainment.

### Demographics and Comorbidities

Analyses were performed using covariates assessed in the BEST-CLI (Best Endovascular Versus Best Surgical Therapy in Patients With Critical Limb Ischemia) randomized clinical trial^12^ (eTable 2 in Supplement 2). Specifically, age was defined at the time of the index procedure. Sex was self-reported from fixed categories of female and male. Race and ethnicity were assessed because PAD is often diagnosed at more advanced stages (eg, CLTI) in Black and Hispanic patients, who also experience greater functional decline and higher rates of PAD-related amputation.^13^ Race was based on self-reported race and country of origin, selecting from race categories of American Indian or Alaska Native, Asian, Black or African American, Native Hawaiian or Other Pacific Islander, White, more than 1 race, unknown, or other and collapsed into Black or African American, White, and other, given low sample size prohibiting more granular detail. Ethnicity was also self-reported and defined as Hispanic and non-Hispanic. Relevant comorbidities were defined in the eMethods in Supplement 1. All participants with missing comorbidity and demographic data were less than 10% of each dataset, including 367 of 4841 patients who underwent open intervention and 206 of 18 060 patients who underwent endovascular intervention (eFigure 1 in Supplement 1). These participants with missing data were excluded from analysis.

### Statistical Analysis

Descriptive statistics were performed to calculate the distribution of all demographic and clinical variables across index procedure type, and differences were analyzed using χ^2^ and *t* tests (eTable 3 in Supplement 2). Cox proportional hazards regression models were used to quantify the association between index procedure type and conduit types with time to adverse events, adjusting for relevant covariates. An interaction *P* value was calculated to identify significant interactions between these demographic subgroups and the index procedure or conduit type potentially associated with the risk of adverse events.

To reduce confounding errors and imbalance, open interventions were propensity matched with endovascular interventions by using the following covariates: age, sex, and self-reported race, ethnicity, body mass index (BMI; calculated as weight in kilograms divided by height in meters squared) smoking status, hypertension, diabetes, coronary artery disease, congestive heart failure, stroke, chronic obstructive pulmonary disease, chronic kidney disease, statin medication, antiplatelet agent, and anticoagulation medication. Success of nearest-neighbor matching was confirmed by comparing standardized mean differences in a love plot (eFigure 2 in Supplement 1). Likelihood ratio tests were performed to compare models when analyzing categories within GSV conduit type and prosthetic type. Statistical significance was defined as a 2-sided *P* < .05. Statistical analysis was performed using R, version 4.4.1 (R Project for Statistical Computing).

## Results

### Demographic Characteristics

A total of 22 328 patients (8056 [36.1%] female, 11 283 [50.5%] male; 197 [0.9%] Asian, 3517 [15.8%] Black or African American, 17 547 [78.6%] White, and 1067 [4.8%] other race) with claudication who underwent an index revascularization procedure were included in this analysis (eFigure 1 in Supplement 1). The median (IQR) length of follow-up for 4474 patients who underwent an index open surgical bypass was 1.0 (0.3-1.2) years (maximum, 8.3 years). The median (IQR) length of follow-up for 17 854 patients who underwent an index endovascular intervention was 1.0 (0.9-1.4) years (maximum, 6.5 years). The majority of patients were not obese (BMI >30, 1589 [35.6%] for open vs 6284 [35.2%] for endovascular; *P* = .14), White (3792 [84.8%] for open vs 13 755 [77.0%] for endovascular; *P* < .001) and male (3334 [74.5%] for open vs 10 952 [61.3%] for endovascular; *P* < .001) with comparable ages (mean [SD] age, 64.4 [10.0] years for open vs 68.6 [10.2] years for endovascular; mean [SD] age for the entire cohort, 67.7 [10.3] years) (Table). Many patients were prior smokers (1933 [43.2%] for open vs 8082 [45.3%] for endovascular; *P* < .001) and did not have diabetes (2968 [66.3%] for open vs 9910 [55.5%] for endovascular; *P* < .001). Cardiovascular comorbidities were prevalent in both index procedure types (eg, hypertension, 3693 [82.5%] for open and 15 750 [88.2%] for endovascular). Most patients were receiving statin (3244 [72.5%] for open vs 13545 [75.9%] for endovascular) and aspirin (3270 [73.1%]) for open vs 13 015 [72.9%] for endovascular) medications. There were more patients receiving direct oral anticoagulants (320 [7.2%] for open vs 1789 [10.0%] for endovascular; *P* < .001) than warfarin (225 [5.0%] for open vs 584 [3.3%] for endovascular; *P* < .001). With regards to operative characteristics, endovascular interventions most commonly targeted superficial femoral artery (SFA) lesions at 60.2% (12 016 SFA and 3568 SFA plus popliteal lesions of 25 868 lesions treated), which occurred in 94.1% of endovascular procedures. A total of 4474 open surgical bypass procedures most often used the common femoral artery as the graft origin (3250 [72.6%]) and above the knee popliteal artery as the graft recipient (2263 [50.6%]) as compared with below the knee popliteal artery graft recipients (2211 [49.4%]) (eTable 4 in Supplement 1).

**Table.** Demographics Stratified by Open Surgical Bypass Compared With Endovascular Intervention for Initial Treatment of Claudication

Characteristic	Patients, No. (%)		*P* value
	Open surgical infrainguinal bypass (n = 4474)	Endovascular intervention (n = 17 854)	
Age, mean (SD),y	64.4 (10.0)	68.6 (10.2)	<.001
Age categories, y			
<65	2226 (49.8)	6030 (33.8)	<.001
65-75	1655 (37.0)	7210 (40.4)	
>75	593 (13.3)	4614 (25.8)	
Sex			
Female	1140 (25.5)	6916 (38.7)	<.001
Male	3334 (74.5)	10 952 (61.3)	
Laterality			
Right	2209 (49.4)	9047 (50.7)	.12
Left	2265 (50.6)	8807 (49.3)	
BMI category^a^			
<18.5	88 (2.0)	377 (2.1)	.14
18.5-25.0	1088 (24.3)	4642 (26.0)	
25.0-30.0	1709 (38.2)	6551 (36.7)	
30.0-40.0	1448 (32.4)	5694 (31.9)	
>40.0	141 (3.2)	590 (3.3)	
Race			
Asian	15 (0.3)	182 (1.0)	<.001
Black or African American	522 (11.7)	2995 (16.8)	
White	3792 (84.8)	13 755 (77.0)	
Other^b^	145 (3.2)	922 (5.2)	
Hispanic ethnicity	151 (3.4)	992 (5.6)	<.001
Hypertension	3693 (82.5)	15 750 (88.2)	<.001
Diabetes			
None	2968 (66.3)	9910 (55.5)	<.001
Diet controlled	174 (3.9)	682 (3.8)	
Noninsulin medication	726 (16.2)	3666 (20.5)	
Insulin dependent	606 (13.5)	3596 (20.1)	
Smoking			
Never	513 (11.5)	3220 (18.0)	<.001
Prior	1933 (43.2)	8082 (45.3)	
Current	2028 (45.3)	6552 (36.7)	
CAD			
None	3341 (74.7)	11 819 (66.2)	<.001
Without MI	279 (6.2)	1976 (11.1)	
With MI	854 (19.1)	4059 (22.7)	
PCI	817 (18.3)	4310 (24.1)	<.001
CABG	623 (13.9)	3045 (17.1)	<.001
CHF	424 (9.5)	2451 (13.7)	<.001
Stroke	7 (0.2)	2176 (12.2)	<.001
COPD	1155 (25.8)	4287 (24.0)	.01
Creatinine, mean (SD), mg/dL	1.04 (0.49)	1.07 (0.50)	<.001
eGFR category, mL/min/1.73 m^2^			
>60	3433 (76.7)	11 878 (66.5)	<.001
30-60	906 (20.3)	5063 (28.4)	
<30	135 (3.0)	913 (5.1)	
Dialysis	38 (0.8)	396 (2.2)	<.001
Dependent ambulation	423 (9.5)	1922 (10.8)	.007
ASA Class			
0	0	4508 (25.2)	<.001
1	31 (0.7)	13 007 (72.9)	
2	384 (8.6)	274 (1.5)	
3	3524 (78.8)	65 (0.4)	
4	535 (12.0)	0	
Statin use			
No	1123 (25.1)	3571 (20.0)	<.001
Yes	3244 (72.5)	13 545 (75.9)	
No for medical reasons	83 (1.9)	644 (3.6)	
Nonadherent	24 (0.5)	94 (0.5)	
Aspirin use			
No	1047 (23.4)	4513 (25.3)	<.001
Yes	3270 (73.1)	13 015 (72.9)	
No for medical reasons	119 (2.7)	275 (1.5)	
Nonadherent	38 (0.8)	51 (0.3)	
Clopidogrel	892 (19.9)	5174 (29.0)	<.001
Prasugrel	24 (0.5)	128 (0.7)	.23
Ticagrelor	19 (0.4)	308 (1.7)	<.001
DOAC	320 (7.2)	1789 (10.0)	<.001
Warfarin	225 (5.0)	584 (3.3)	<.001
Length of hospital stay, mean (SD), d	4.1 (13.8)	1.9 (78.7)	.07

Abbreviations: ASA, American Society of Anesthesiologists; BMI, body mass index; CABG, coronary artery bypass graft; CAD, coronary artery disease; CHF, congestive heart failure; COPD, chronic obstructive pulmonary disease; DOAC, direct oral anticoagulant; eGFR, estimated glomerular filtration rate; MI, myocardial infarction; PCI, percutaneous coronary intervention.

SI conversion factor: to covert creatinine to micromoles per liter, multiply by 88.4.

^a^ BMI category calculated as weight in kilograms divided by height in meters squared.

^b^ Other race included American Indian or Alaska Native, Native Hawaiian or other Pacific Islander, more than 1 race, and unknown or other.

### Comparison of Endovascular With Open Procedures

The overall rate of total amputations was significantly higher in the endovascular group compared with the open group (304 [1.7%] for endovascular vs 65 [1.5%] for open; *P* < .001), although the absolute differences were small (eTable 5 in Supplement 1). Notably, minor amputations were more common in the endovascular group (163 [0.9%] vs 18 [0.4%]), while major amputations did not significantly differ between the groups (141 [0.8%] vs 47 [1.1%]; *P* = .11). Specifically, below the knee amputation occurred slightly more frequently in the endovascular group (98 [0.5%] vs 20 [0.4%]), while above the knee amputation was more common in the open group (27 [0.6%] vs 43 [0.2%]). The mean (SD) time to major amputation was significantly shorter in the endovascular group (195.0 [185.9] days vs 434.6 [291.6] days; *P* < .001). Procedures were stratified by amputation status to better understand anatomic lesion classifications (eTable 6 in Supplement 1). In the endovascular cohort, severe distal lesions classified as TransAtlantic Inter-Society Consensus Document lesions 2, 4, and 5 were significantly more common for major amputations compared with participants without amputations. No meaningful anatomical risk stratification could be performed in the open bypass cohort due to the absence of Global Limb Anatomic Staging System classification data.

**Figure 1.**
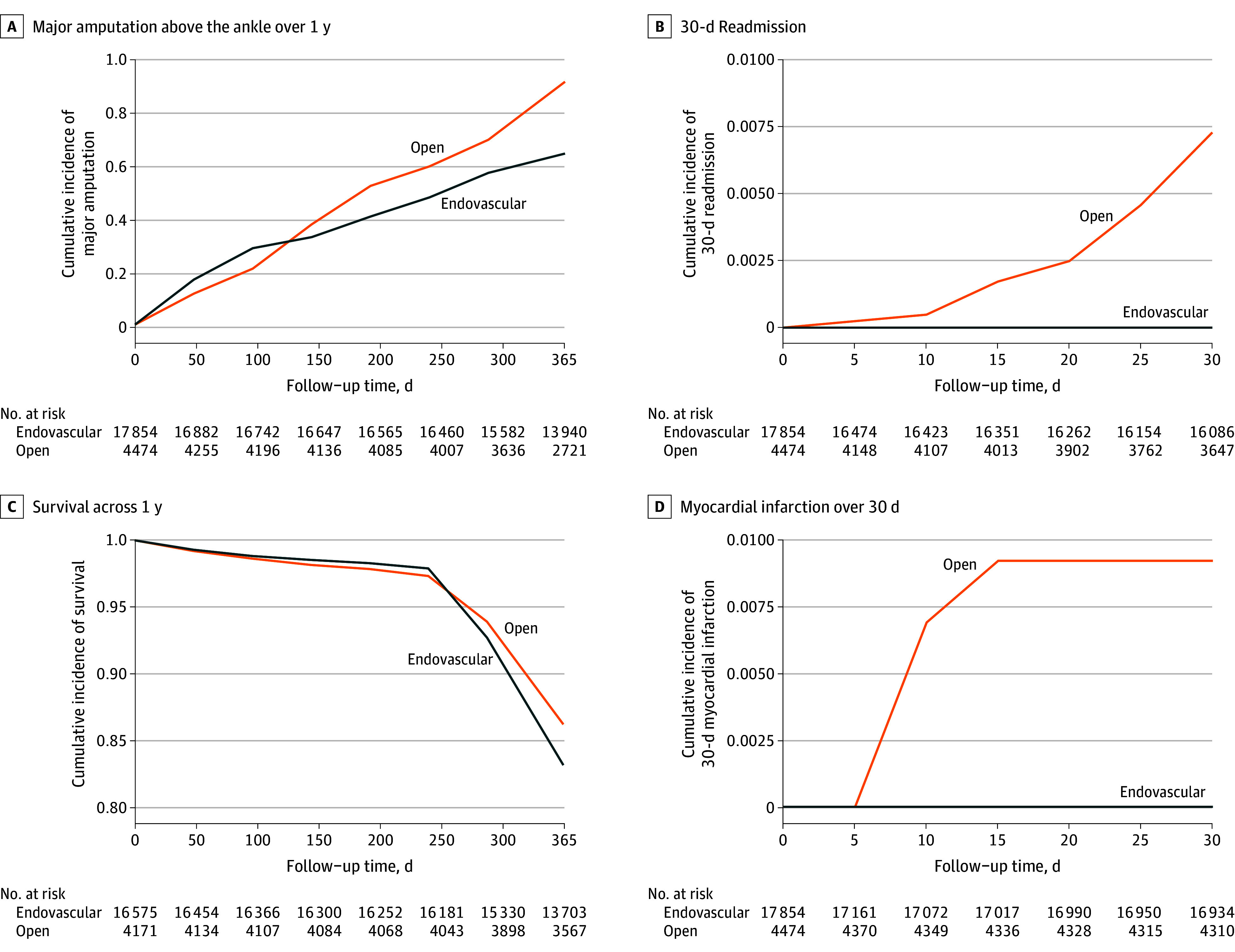
Kaplan-Meier Curves of Vascular Outcomes Stratified by Index Open Surgical Bypass (Open) and Endovascular Interventions for Initial Treatment of Claudication

**Figure 2.**
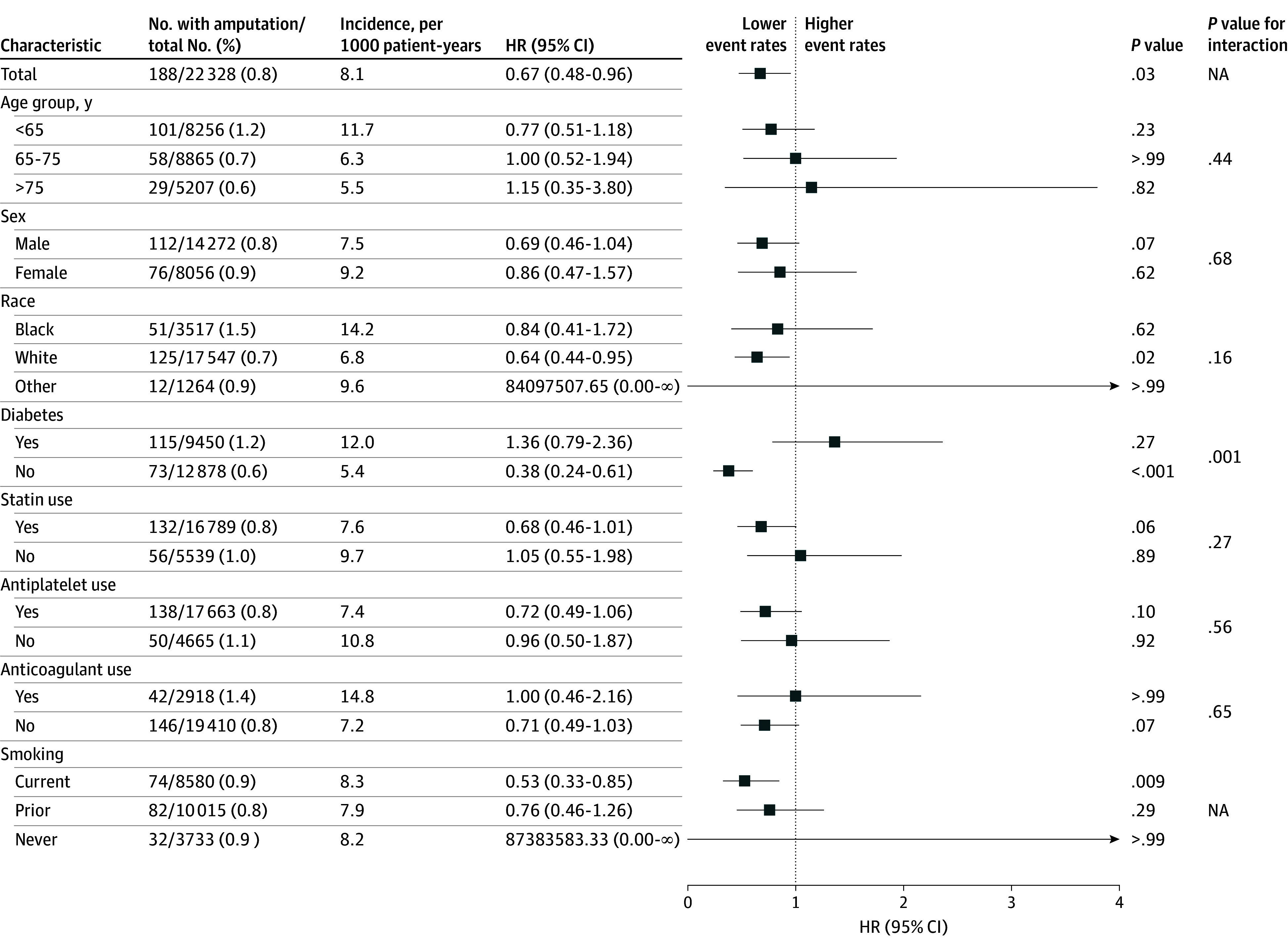
Risk of 1-Year Major Amputation After Open Infrainguinal Procedures vs Endovascular Intervention, by Clinically Relevant Subgroups The reference category intervention was index open infrainguinal procedures. Hazard ratios (HRs) with corresponding 95% CIs are reported based on Cox proportional hazards regression models with covariates listed in the Methods. Subgroup analyses were performed by splitting cohorts based on presence or absence of relevant subgroups. An interaction *P* value was calculated to identify significant interactions between subgroups and index procedure type influencing amputation risk. NA indicates not analyzed.

The cumulative incidence of major amputation at 1 year was lower in the endovascular group at 0.6% compared with the open surgical bypass group at 0.9% (Figure 1). After adjustment for relevant cardiovascular comorbidities, there was an association between endovascular intervention and lower risk of major amputation within 1 year compared with open surgical bypass, with a hazard ratio (HR) of 0.67 (95% CI, 0.48-0.96; *P* = .03) (Figure 2). At the 1-year period, the risk of major amputation was significantly lower among patients without diabetes (HR, 0.38 [95% CI, 0.24-0.61]; *P* < .001) compared with patients with diabetes (HR, 1.36 [95% CI, 0.79-2.36]; *P* = .27) (*P* = .001 for interaction). To ensure results were not reflecting patient comorbidities, we performed propensity matching that showed the association remained, with a lower 1-year major amputation risk after endovascular intervention (HR, 0.53 [95% CI, 0.33-0.84]; *P* = .007) and a higher hazard among patients with diabetes (HR, 0.96 [95% CI, 0.49-1.89]) (*P* = .003 for interaction) (eFigure 3 in Supplement 1).

Secondary outcomes included death, 30-day readmission, and myocardial infarction. The risk of death was significantly higher following endovascular intervention compared with open surgical bypass at an HR of 2.09 (95% CI, 1.97-2.23; *P* < .001), which was attenuated after matching at an HR of 1.67 (95% CI, 1.54-1.90; *P* < .001) (eFigure 4 in Supplement 1). There was an association between endovascular procedures and lower risk of 30-day readmission compared with open procedures (HR, 0.24 [95% CI, 0.08-0.81]; *P* = .004), with no significant differences observed among demographic groups (eFigure 5 in Supplement 1). After matching, the sample size for the outcome of 30-day readmission and 30-day myocardial infarction was too small for analysis, and the models did not converge.

### Conduit-Related Outcomes

**Figure 3.**
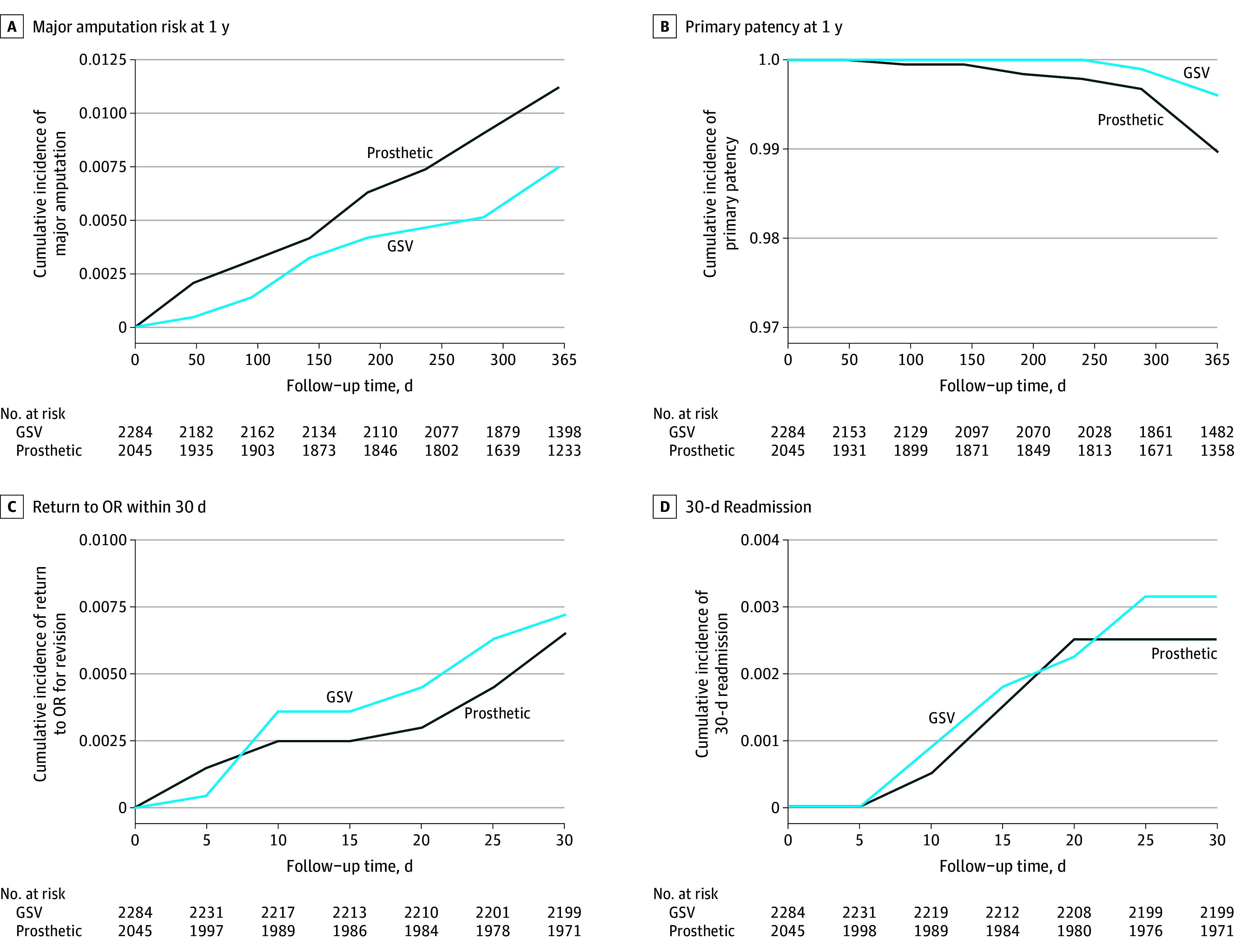
Kaplan-Meier Curves of Vascular Outcomes Stratified by Great Saphenous Vein (GSV) and Prosthetic Conduit Approaches for Initial Treatment of Claudication OR indicates operating room.

**Figure 4.**
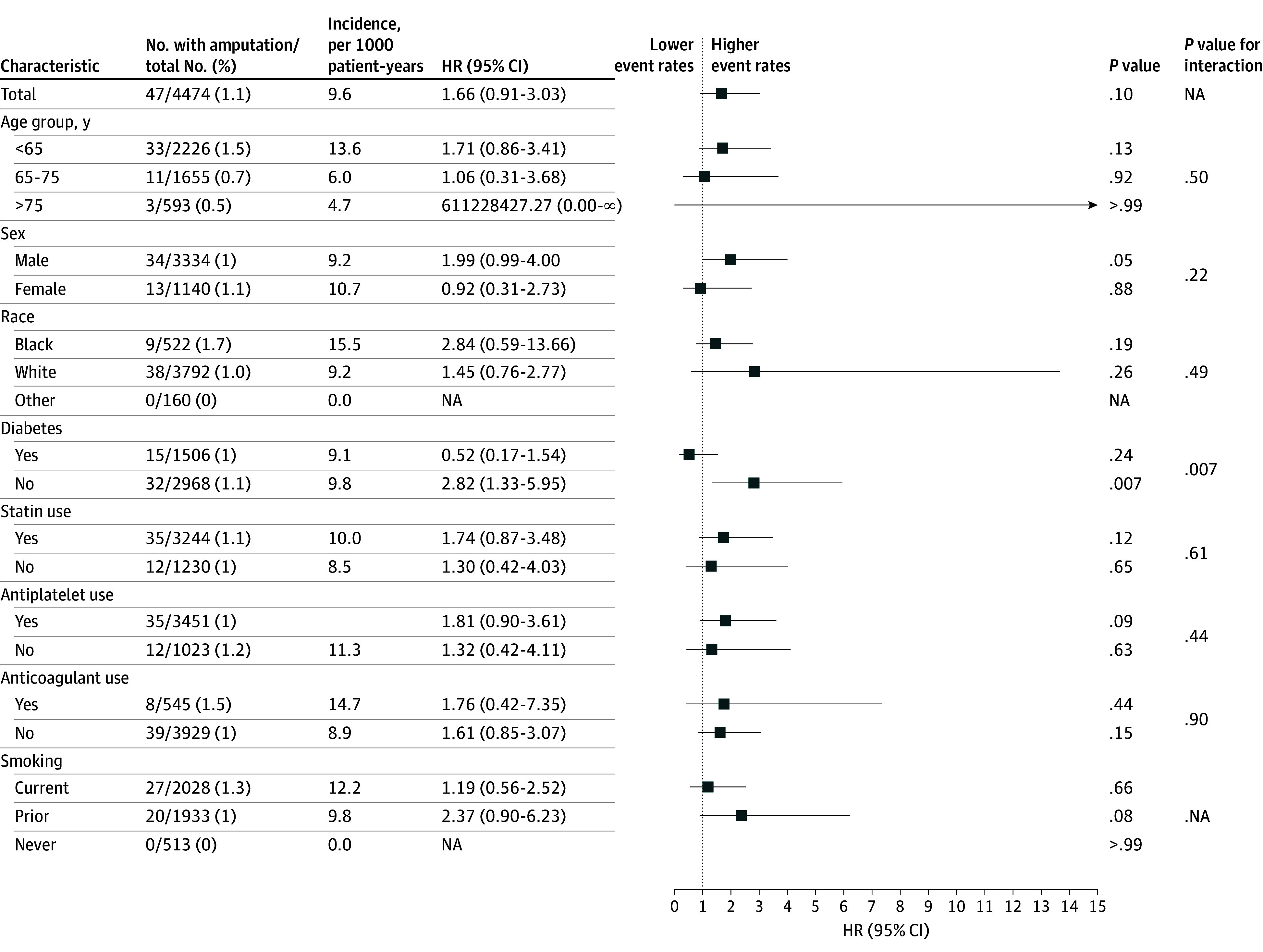
Risk of Major Amputation After 1 Year Using Prosthetic Conduit Compared With Great Saphenous Vein Approaches for Initial Treatment of Claudication, by Clinically Relevant Subgroups The reference category conduit was great saphenous vein. Hazard ratios (HRs) with corresponding 95% CIs are reported based on Cox proportional hazards regression models with covariates indicated in the Methods. Subgroup analyses were performed by splitting cohorts based on presence or absence of the clinical subgroup. An interaction *P* value was calculated to identify significant interactions between subgroups and intervention category influencing amputation and primary patency risk. NA indicates not analyzed.

Subgroup analysis was performed to compare 2284 patients with a GSV conduit and 2045 patients with a prosthetic conduit, excluding any composite conduits (eTable 7 in Supplement 1). At 1 year, the prosthetic group had a higher cumulative incidence of major amputation (prosthetic, 1.1% vs GSV, 0.8%), lower primary patency (prosthetic, 99.0% vs GSV, 99.6%), but fewer 30-day returns to the operating room (prosthetic, 0.3% vs GSV, 1.8%) and hospital readmission (prosthetic, 0.2% vs GSV, 0.3%) compared with the GSV group (Figure 3). After adjusting for aforementioned covariates in addition to graft recipient, the prosthetic group was not associated with increased risk for a major amputation at 1 year (HR, 1.66 [95% CI, 0.91-3.03]; *P* = .10), whereas patients without diabetes were associated with higher risk of 1-year major amputation compared with those with diabetes (HR, 2.82 [95% CI, 1.33-5.95]; *P* = .007) (Figure 4). The prosthetic group also had a similar primary patency (HR, 0.96 [95% CI, 0.90-1.02]; *P* = .23) at 1 year compared with GSV (eFigure 6 in Supplement 1). Otherwise, the risks of major amputation and primary patency did not differ significantly by other demographic or comorbidity groups studied. Secondary outcomes of 30-day return to the operating room and readmission were both associated with lower rates in the prosthetic group (eFigure 7 in Supplement 1).

Among GSV conduits, the unadjusted cumulative incidence of major amputation at 1 year was highest for reversed GSV, whereas transposed GSV had the highest cumulative incidence of primary patency (eFigure 8 in Supplement 1). However, after adjusting for the aforementioned covariates, reversed GSV was associated with the lowest risk estimate for major amputation after 1 year (HR, 0.40 [95% CI, 0.20-0.83]; *P* = .01) (*P* = .002 for likelihood ratio test) compared with in situ and transposed GSV (likelihood ratio test *P* < .001), which had equivalent outcomes (eFigure 9 in Supplement 1). The primary patency rates were not significantly different compared with non-GSV conduits among all 3 groups. The risk of return to the operating room and readmission in 30 days were similar between GSV conduit groups. When comparing the 2 common options for prosthetic conduits, we found unadjusted cumulative incidence of major amputation at 1 year and primary patency were associated with polyethylene terephthalate grafts (eFigure 10 in Supplement 1). After adjusting for the aforementioned covariates, there was an association between polyethylene terephthalate grafts and a higher risk of major amputation at 1 year (HR, 4.78, [95% CI, 1.02-22.30]; *P* = .047) compared with PTFE (HR 1.59 [95% CI, 1.15-2.20]; *P* = .005) (likelihood ratio test *P* = .002). Confidence intervals were wide, and sample sizes were too small to draw reliable conclusions for other outcomes.

## Discussion

In this retrospective cohort study of 22 328 patients with PAD with claudication who underwent an index revascularization procedure, endovascular procedures were associated with lower risk of major amputation but higher risk of mortality at 1 year compared with open surgical bypass procedures. Among patients who underwent open surgical bypass, prosthetic conduits showed a higher cumulative incidence of major amputation than GSV conduits but were not associated with the adjusted increased risk of major amputation at 1 year. Prosthetic conduits and GSV conduits had similar rates of primary patency at 1 year. GSV configurations had varying risks of adverse outcomes, with reversed GSV configurations having the lowest risk of major amputation, while primary patency was similar for all 3 groups. The prosthetic graft type of polyethylene terephthalate compared with PTFE showed significantly higher major amputation risk at 1 year. These data suggest that patients with claudication may benefit from an endovascular approach, and if an open approach is needed, reversed GSV conduits should be prioritized in surgical bypass.

The BEST-CLI and BASIL (Bypass versus Angioplasty in Severe Ischaemia of the Leg) trials evaluated outcomes of open bypass vs endovascular intervention in patients with CLTI.^14,15^ While BASIL found no significant difference between the 2 strategies within the first year and a benefit to open bypass in the second year, the BEST-CLI trial showed higher amputation risk with endovascular treatment among patients with adequate GSV.^14^ In contrast, endovascular-first strategies remain standard for claudication.^8^ However, comparing these approaches is difficult to quantify given the heterogeneity of endovascular techniques to treat complex multistaged lesions^16^ in a considerably more frail population.^17^ Prior observational studies have shown low and similar in-hospital amputation rates,^18^ but long-term data remain limited and inconsistent.^19^

In our larger study of 22 328 patients with claudication undergoing femoropopliteal index interventions, we observed approximately 30% lower risk of major amputation at 1 year (HR, 0.67 [95% CI, 0.48-0.96]) among patients treated with endovascular intervention propensity matched for clinical and demographic variables, supporting the continued use of an endovascular-first approach in appropriately selected patients with claudication. These differences from the BASIL and BEST-CLI trials may reflect that patients with claudication have preserved limb viability and a more heterogeneous disease course that may benefit from earlier endovascular treatment.^15^ Although matching reduced baseline differences, the risk of death remained significantly higher in the endovascular group, suggesting that patients selected for endovascular treatment may still represent a sicker population overall.^18^ In our subgroup analysis, diabetes was associated with a higher amputation risk, even after matching, underscoring the vulnerability of patients with diabetes to more aggressive disease.

GSV is generally regarded as a more durable graft than prosthetic options^20^; however, evidence comparing GSV configurations is limited.^21^ Even with a large sample size and adjustment for cardiovascular comorbidities, our study showed GSV conduits were not associated with a decreased 1-year major amputation risk (HR, 1.66 [95% CI, 0.91-3.03]; *P* = .10) and had primary patency rates similar to prosthetic conduits. We also found that reversed GSV was associated with the lowest risk of major amputation compared with in situ or transposed GSV configurations. This finding is consistent with a meta-analysis of 37 studies specific to femoropopliteal bypass only, which found that reversed GSV had significantly lower failure rates compared with in situ configurations.^22^ Similarly, a prospective randomized clinical trial found cumulative limb salvage rates were higher for reversed GSV at 87% (n = 80) compared with in situ grafts at 78% (n = 82), although the difference was not statistically significant.^23^ We also found no significant differences in 1-year primary patency across GSV configurations, consistent with earlier VQI data (2003 to 2021) showing similar outcomes between reversed and nonreversed GSV bypasses.^24^

Varying outcomes related to GSV configuration may reflect complex decision-making regarding GSV configuration, which is highly dependent on the patient’s anatomy, quality of the vein, and surgeon experience.^25^ Whereas some studies have evaluated the impact of configuration, most have focused on vein diameter, as smaller diameters are strongly associated with early graft failure, as noted in the PREVENT III (Project or Ex-Vivo Vein Graft Engineering via Transfection III) trial for patients with CLTI.^26^ Unfortunately, graft diameter was not available in our dataset, limiting our ability to assess its effect. There are theoretical advantages and disadvantages to different GSV configurations. Reversed GSV grafts do not require valvulotomy, potentially reducing the risk of injury, but may pose challenges due to size mismatch at the anastomotic sites. In contrast, in situ grafts provide better size matching but require valvulotomy, which may injure the vein wall and compromise long-term durability.^27^ Therefore, the SVS guidelines indicate that both reversed and in situ GSV grafts are acceptable options.^8^ PTFE grafts have previously been shown to have greater primary patency over polyethylene terephthalate grafts in CLTI patients.^28^ The durability of PTFE grafts has been attributed to lower thrombogenicity compared with polyethylene terephthalate and better flow characteristics in smaller-caliber vessels of the lower extremities.^29,30^ Our results support these findings, showing the use of polyethylene terephthalate grafts associated with a higher risk estimate for major amputation after 1 year compared with PTFE.

### Limitations

This study has limitations that warrant consideration when interpreting the results. The absence of detailed lesion classification data limited our ability to stratify outcomes based on the anatomical complexity of the treated vascular disease. However, we used data on bypass graft origin, bypass graft recipient, and arteries treated during endovascular intervention to classify femoropopliteal disease patterns as accurately as possible. We were unable to account for variations in surgeon technique or experience. For patients who underwent endovascular treatment, we lacked information on the availability of suitable veins for potential bypass procedures, potentially confounding the comparison between endovascular and open surgical approaches by indication. Furthermore, the quality of veins used in bypass procedures could not be assessed.

The definition of many outcomes in the VQI were inconsistent across infrainguinal and PVI datasets, potentially affecting the accuracy of these complication rates. Procedures performed at outside institutions were not recorded in this database, potentially leading to misidentification of index procedures. Patient adherence to prescribed medications was not accounted for in this study. Additionally, we did not capture changes in outcomes over time, which may have disproportionately impacted endovascular procedures due to rapid technological advancements. The VQI data used in this study may have incomplete long-term outcomes, such as 1-year major amputation and primary patency rates. These patency rates were ascertained from clinical practice, and therefore variation in institutional follow-up practices may have influenced the timing and accuracy of patency reporting.

This was a complete case analysis, limited by missing comorbidity and follow-up data, which led to the exclusion of a substantial number of patients. The study did not incorporate outcomes from the VQI Vascular Implant Surveillance and Interventional Outcomes Network project, which could have provided additional valuable data for analysis but would have lacked information on procedure laterality and associated amputation outcomes. The outcomes derived from the VQI Long-Term Follow-Up dataset did not include Rutherford grades of ischemia. These limitations highlight the need for cautious interpretation of the results and underscore areas for improvement in future studies. Despite these constraints, our findings provide valuable insights into the comparative outcomes of open surgical bypass and endovascular interventions in patients with claudication.

## Conclusions

In this retrospective cohort study of data derived from the VQI database, we compared index endovascular procedures and open surgical bypass, including conduit types, among patients with claudication to assess the risk of major amputation. We showed that endovascular procedures were associated with a lower risk of major amputation at 1 year compared with open surgical bypass after adjusting for demographics, comorbidities, and medications. Among patients who underwent open surgical bypass, reversed GSV conduits were associated with the lowest risk of major amputation at 1 year, while prosthetic conduits and in situ GSV configurations had equivalent outcomes. Patients with claudication may benefit most from endovascular-first intervention and subsequent open bypass using reversed GSV conduits when available.

## Notes

Supplement 1.
eMethods.
**eTable 1.** Ankle brachial indices (ABI) and toe pressures (TP) in each cohort **eTable 4.** Operative characteristics in both open infrainguinal (INFRA) and endovascular peripheral vascular intervention (PVI) data sets **eTable 5.** Outcomes stratified by open infrainguinal (INFRA) and endovascular peripheral vascular intervention (PVI) data sets **eTable 6.** Anatomic lesion classification in both open infrainguinal (INFRA) and endovascular peripheral vascular intervention (PVI) data sets **eTable 7.** Demographics within the open infrainguinal bypass vascular quality initiative data set stratified by graft saphenous vein and prosthetic conduit for initial treatment of claudication **eFigure 1.** Flowchart of inclusion and exclusion of index endovascular and open procedures for comparative analysis **eFigure 2.** Love plot illustrating standardized mean differences in patient comorbidities before and after propensity score matching of patients undergoing endovascular procedures to 4,474 patients undergoing open procedures for claudication **eFigure 3.** Risk of adverse outcomes in propensity matched endovascular compared to open intervention by clinically relevant subgroups **eFigure 4.** Risk of major amputation and death using endovascular compared to open intervention by demographic subgroups **eFigure 5.** Risk of readmission and myocardial infarction using endovascular compared to open intervention by demographic subgroups **eFigure 6.** Risk of major amputation and primary patency after one year using prosthetic conduit compared to great saphenous vein for initial treatment of claudication by clinically relevant subgroups **eFigure 7.** Risk of outcomes using prosthetic conduit compared to great saphenous vein for initial treatment of claudication stratified by demographic subgroups **eFigure 8**. Risk of adverse outcomes stratified by GSV configuration type for initial treatment of claudication **eFigure 9.** Risk of adverse outcomes stratified A) great saphenous vein and B) prosthetic conduit type for initial treatment of claudication by demographic subgroups **eFigure 10.** Risk of adverse outcomes stratified by prosthetic conduit type for initial treatment of claudication

Supplement 2. **eTable 2.** Definition of demographic and comorbidities before intervention in open infrainguinal (INFRA) and endovascular peripheral vascular intervention (PVI) in Vascular Quality Initiative (VQI) data sets **eTable 3.** Definition of outcomes during admission in open infrainguinal (INFRA) and endovascular peripheral vascular intervention (PVI) data sets

Supplement 3.
Data Sharing Statement
