# Correction to Author Degree

**DOI:** 10.1001/jamanetworkopen.2025.49614

**Published:** 2025-11-25

The Original Investigation titled “Endovascular Therapy, Open Surgical Bypass, and Conduit Types for Index Treatment of Claudication,”^1^ published on October 16, 2025, was corrected to fix the degree for Gabriel Jabbour from BS to MD.
