# Supplementary material for: Endovascular Therapy, Open Surgical Bypass, and Conduit Types for Index Treatment of Claudication
Source: JAMA Netw Open. 2025 Oct 16;8(10):e2533352. doi: 10.1001/jamanetworkopen.2025.33352 (PMC12531885; doi:10.1001/jamanetworkopen.2025.33352)
Supplement: Supplement 1. — eMethods. eTable 1. Ankle brachial indices (ABI) and toe pressures (TP) in each cohort eTable 4. Operative characteristics in both open infrainguinal (INFRA) and endovascular peripheral vascular intervention (PVI) data sets eTable 5. Outcomes stratified by open infrainguinal (INFRA) and endovascular peripheral vascular intervention (PVI) data sets eTable 6. Anatomic lesion classification in both open infrainguinal (INFRA) and endovascular peripheral vascular intervention (PVI) data sets eTable 7. Demographics within the open infrainguinal bypass vascular quality initiative data set stratified by graft saphenous vein and prosthetic conduit for initial treatment of claudication eFigure 1. Flowchart of inclusion and exclusion of index endovascular and open procedures for comparative analysis eFigure 2. Love plot illustrating standardized mean differences in patient comorbidities before and after propensity score matching of patients undergoing endovascular procedures to 4,474 patients undergoing open procedures for claudication eFigure 3. Risk of adverse outcomes in propensity matched endovascular compared to open intervention by clinically relevant subgroups eFigure 4. Risk of major amputation and death using endovascular compared to open intervention by demographic subgroups eFigure 5. Risk of readmission and myocardial infarction using endovascular compared to open intervention by demographic subgroups eFigure 6. Risk of major amputation and primary patency after one year using prosthetic conduit compared to great saphenous vein for initial treatment of claudication by clinically relevant subgroups eFigure 7. Risk of outcomes using prosthetic conduit compared to great saphenous vein for initial treatment of claudication stratified by demographic subgroups eFigure 8. Risk of adverse outcomes stratified by GSV configuration type for initial treatment of claudication eFigure 9. Risk of adverse outcomes stratified A) great saphenous vein and B) prosthetic conduit type f [file jamanetwopen-e2533352-s001.pdf]

Supplementary Online Content

Bellomo TR, Jabbour G, Manchella M, et al. Endovascular therapy, open surgical bypass, and conduit types for index treatment of claudication. *JAMA Netw Open*. 2025;8(10):e2533352. doi:10.1001/jamanetworkopen.2025.33352

eMethods.
eTable 1. Ankle brachial indices (ABI) and toe pressures (TP) in each cohort
eTable 4. Operative characteristics in both open infrainguinal (INFRA) and endovascular peripheral vascular intervention (PVI) data sets
eTable 5. Outcomes stratified by open infrainguinal (INFRA) and endovascular peripheral vascular intervention (PVI) data sets
eTable 6. Anatomic lesion classification in both open infrainguinal (INFRA) and endovascular peripheral vascular intervention (PVI) data sets
eTable 7. Demographics within the open infrainguinal bypass vascular quality initiative data set stratified by graft saphenous vein and prosthetic conduit for initial treatment of claudication
eFigure 1. Flowchart of inclusion and exclusion of index endovascular and open procedures for comparative analysis
eFigure 2. Love plot illustrating standardized mean differences in patient comorbidities before and after propensity score matching of patients undergoing endovascular procedures to 4,474 patients undergoing open procedures for claudication
eFigure 3. Risk of adverse outcomes in propensity matched endovascular compared to open intervention by clinically relevant subgroups
eFigure 4. Risk of major amputation and death using endovascular compared to open intervention by demographic subgroups
eFigure 5. Risk of readmission and myocardial infarction using endovascular compared to open intervention by demographic subgroups
eFigure 6. Risk of major amputation and primary patency after one year using prosthetic conduit compared to great saphenous vein for initial treatment of claudication by clinically relevant subgroups
eFigure 7. Risk of outcomes using prosthetic conduit compared to great saphenous vein for initial treatment of claudication stratified by demographic subgroups
eFigure 8. Risk of adverse outcomes stratified by GSV configuration type for initial treatment of claudication
eFigure 9. Risk of adverse outcomes stratified A) great saphenous vein and B) prosthetic conduit type for initial treatment of claudication by demographic subgroups
eFigure 10. Risk of adverse outcomes stratified by prosthetic conduit type for initial treatment of claudication
eReferences.

© 2025 Bellomo TR et al. *JAMA Network Open.*

This supplementary material has been provided by the authors to give readers additional information about their work.

© 2025 Bellomo TR et al. *JAMA Network Open*.

e**Methods.**

Exclusion and inclusion criterion within the Vascular Quality Initiative (VQI) data sets

The inclusion criteria specific to the INFRA dataset required the graft origin to be above the knee and the recipient vessel to be the popliteal artery, as aortoiliac disease was not the target lesion for this study group and tibial bypass is not considered an ideal treatment for claudication. Both above and below the knee bypasses were eligible for included. The inclusion criterion specific to the PVI dataset were isolated endovascular procedures and interventions that included treatment of at least one of the following arteries: common femoral, profunda, SFA, or popliteal artery, as these lesions can also likely be treated using bypass grafts. The exclusion criterion included Percutaneous Transmural Arterial Bypass (PTAB) and Deep Venous Arterialization (DVA) procedures, given these hybrid procedures aim to restore arterial blood flow through vein pressurization and are not considered an ideal treatment for claudication. Concomitant femoral endarterectomies were also excluded given these are open procedures.

VQI-Based Definition of Claudication

Within the PVI data set, procedure laterality was defined using PROCSIDE_1, PROCSIDE_2, PROCSIDE_3, and PROCSIDE_4. Symptom status was then assessed using the LEGSYMP_R and LEGSYMP_L variables, where claudication was defined by the values 1, 2, 9 or 10. A value of 1 represented a combined category encompassing mild, moderate, and severe claudication. However, this classification was retired as of 09/22/2016 and has since been replaced with more granular definitions of claudication. A value of 9 represented mild claudication, which the VQI defines as the following: “Although unable to perform some

© 2025 Bellomo TR et al. *JAMA Network Open*.

activities; everyday activities are not limited. Experiences ischemic limb muscle pain that does not limit walking or limits walking only after > 2 blocks (>600 feet or 2 football fields).". A value of 2 represented moderate claudication, which the VQI defines as the following: "Only able to perform short walking distance for routine errands. Experiences ischemic limb muscle pain that limits walking 1-2 blocks (300-600 feet, or 1-2 football fields).". A value of 10 represented severe claudication, which the VQI defined as the following: "Refrains from routine errands that require walking. Experiences ischemic limb muscle pain that limits walking < 1 block (<300 feet or 1 football field).".

Within the INFRA data set, procedure laterality was first determined using the variable SIDE. Symptom status was then defined using R_INDICATIONR, R_INDICATIONL, INDICATION_OCCLUSIVE_R, and INDICATION_OCCLUSIVE_R. The INDICATION variables represented claudication using the value 1, which the VQI defined as the following: "patient reports pain when walking". However, these variables were retired in 3/29/2023 and replaced with the INDICATION_OCCLUSIVE variables, which provide more specific classification using values 1, 2, and 3. A value of 1 represented mild claudication, which the VQI defined as the following: "Although unable to perform some activities; everyday activities are not limited. Experiences ischemic limb muscle pain that does not limit walking or limits walking only after > 2 blocks (>600 feet or 2 football fields).". The value of 2 represented moderate claudication, which the VQI defined as the following: "Only able to perform short walking distance for routine errands. Experiences ischemic limb muscle pain that limits walking 1-2 blocks (300-600 feet, or 1-2 football fields).". The value of 3 represented severe claudication, which the VQI defined as the following: "Refrains from routine errands that require walking. Experiences ischemic limb muscle pain that limits walking < 1 block (<300 feet or 1 football field)."

© 2025 Bellomo TR et al. *JAMA Network Open*.

While ankle-brachial index (ABI) and toe pressure measurements would have been ideal to include in this analysis, a substantial proportion of data was missing—over 24% for each measurement in both the open and endovascular intervention groups (eTable 1). Among the available data, the mean ABIs recorded pre-operatively for endovascular procedures were 0.80 with a toe pressure of 47 mmHg, and for open procedures were 0.73 with a toe pressure of 46 mmHg and 24 mmHg. These measurements are in line with the Society for Vascular Surgery guidelines of claudication[1,2]. Exercise data were also largely missing, with over 93% of fields incomplete in both datasets, and therefore were not included in this analysis.

Definitions of outcomes within the Vascular Quality Initiative (VQI) data sets

Myocardial Infarction (MI) was defined as either a positive troponin level or EKG changes indicative of myocardial ischemia. Death was assessed one year after procedure date. Readmission was defined as a subsequent admission related to the index procedure within 30 days of index procedure.

We examined two additional adverse outcomes specific to the INFRA or open surgical bypass data set only: primary patency was defined as graft surveillance indicating the graft remained patent without any reintervention, reintervention for thrombosis, or thrombosis with loss of patency. Patency was defined using the LTF data field LTF_CURRPATENCY, where the data field indicates patency was determined by doppler, palpable graft pulse, palpable distal pulse, ABI or Duplex Ultrasound at the time of examination. The value of 1 initially indicated primary patency. However, this definition was retired as of 11/03/2022 and replaced with a broader definition of patency. A value of 6 represented patency, which the VQI defines as the following: "The current patency of the bypass is

© 2025 Bellomo TR et al. *JAMA Network Open*.

determined to be open.”. Both values of 1 and 6 were used to indicate patency of the graft. Patency was not assessed in the PVI data set given a high rate of missingness of over 12%. Return to the OR for revision was defined as an additional operation required during the same admission as the index procedure or after discharge within 30 days of the index procedure.

Definitions of comorbidity data within the Vascular Quality Initiative (VQI) data sets

Obesity was categorized using a body mass index (BMI) cutoff of 30 kg/m^2. Current smoking was defined as never smoking, prior smoking, or current smoking at the time of the index procedure. Hypertension was defined as systolic blood pressure ≥140 mmHg, diastolic blood pressure ≥90 mmHg, or if previously recorded in their chart. Diabetes was defined as none, controlled with diet, controlled with non-insulin medications, or controlled with insulin. Coronary artery disease (CAD) was defined as a diagnosis of stable angina, unstable angina, or myocardial infarction. Congestive Heart Failure (CHF) was defined by SNOMED CT classification greater than Class I, which is cardiac disease without resulting limitations of physical activity. Stroke was defined pre-operatively as minor with non-disabling limitations and major with disabling limitations sufficient to require care assistance. Chronic Obstructive Pulmonary Disease (COPD) was defined as any disease that results in COPD including asthma, chronic bronchitis, emphysema, and asbestosis. Chronic Kidney Disease (CKD) was determined based on estimated glomerular filtration rate calculated using the CKD Epidemiology Collaboration equation. Statin medications were defined as any of the HMG-CoA reductase inhibitors used to reduce cholesterol, taken within 36 hours of the index procedure. Antiplatelet medications were defined as aspirin, clopidogrel, prasugrel, or

© 2025 Bellomo TR et al. *JAMA Network Open*.

ticagrelor taken within one week of the index procedure. Anticoagulant medications were defined as taking a direct oral anticoagulant (DOAC) or warfarin medication prior to the procedure.

© 2025 Bellomo TR et al. *JAMA Network Open*.

eTable 1. Ankle brachial indicies (ABI) and toe pressures (TP) in each cohort<br>*Data field only avalible for the ipsilateral intervention side.								
**Data Field**	**Endovascular** (n=17,854)				**Open** (n=4,474)			
	**Pre-operative**		**Post-operative**		**Pre-operative**		**Post-operative**	
	Mean (SD)	Missingness (%)	Mean (SD)	Missingness (%)	Mean (SD)	Missingness (%)	Mean (SD)	Missingness (%)
**ABI Right**	0.80 (0.84)	4425 (24.78%)	0.80 (0.84)	9261 (51.87%)	0.73 (0.33)	1107 (24.74%)	0.94 (0.29)*	2478 (55.39%)
**ABI Left**	0.80 (0.96)	4448 (24.91%)	0.80 (0.96)	9279 (51.97%)	0.73 (0.31)	1103 (24.65%)	NA	NA
**Toe Pressure Right (mmHg)**	46.93 (42.91)	12281 (68.79%)	46.93 (42.91)	13963 (78.21%)	46.28 (57.55)	4471 (99.93%)	62 (37.26)*	3726 (83.28%)
**Toe Pressure Left (mmHg)**	47.16 (43.45)	12318 (68.99%)	47.16 (43.45)	13956 (78.17%)	24.52 (41.99)	4471 (99.93%)	NA	NA

© 2025 Bellomo TR et al. *JAMA Network Open*.

**eTable 4.** Operative characteristics in both open infrainguinal (INFRA) and endovascular peripheral vascular intervention (PVI) data sets	
**Endovascular peripheral vascular intervention (PVI) (n = 17,854)**	**n (%)**
**Fluoroscopy Time Minutes (mean (SD))**	19.46 (22.12)
**Treated arteries (%)**	**25868 arteries treated**
Anterior Tibial	918 (3.5%)
Tibial Peroneal Trunk	1059 (4.1%)
Posterior Tibial	706 (2.7%)
Peroneal	696 (2.7%)
Dorsal Pedal	28 (0.1%)
Plantar	11 (0.0%)
Internal Iliac	25 (0.1%)
Common iliac	582 (2.2%)
External Iliac	918 (3.5%)
Common + External Iliac	191 (0.7%)
Common femoral	1105 (4.3%)
Profunda	235 (0.9%)
Superficial Femoral Artery	12016 (46.5%)
Popliteal	3810 (14.7%)
Superficial Femoral Artery + Popliteal	3568 (13.8%)
**Open infrainguinal (INFRA) bypass data set (n=4,474)**	**n(%)**
Procedure Time Minutes (mean (SD))	195.46 (92.52)
Grion Incision (%)	
Missing	677 (15.6)

© 2025 Bellomo TR et al. *JAMA Network Open*.

Vertical	2656 (61.2)
Horizontal	1010 (23.3)
Graft Origin (%)	
External Iliac	42 (0.9)
Common Femoral	3250 (72.6)
Profunda	83 (1.9)
Superficial Femoral Artery	756 (16.9)
Above Knee	343 (7.7)
Graft Recipient (%)	
BK popliteal	2211 (49.4)
AK popliteal	2263 (50.6)
Graft Vein Type (%)	
None	2024 (45.5)
Reversed GSV	1409 (31.6)
In Situ GSV	476 (10.7)
Non-reversed Transposed GSV	436 (9.8)
Lesser Saphenous	21 (0.5)
Cephalic	18 (0.4)
Basilic	9 (0.2)
Composite Vein	60 (1.3)
Prosthetic Type (%)	
None	2366 (53.0)
Dacron	63 (1.4)
PTFE	1982 (44.4)
Non-Autologous Biologic	48 (1.1)
Other/Composite Vein	8 (0.2)

© 2025 Bellomo TR et al. *JAMA Network Open*.

eTable 5. Outcomes stratified by open infrainguinal (INFRA) and endovascular peripheral vascular intervention (PVI) data sets

	**Endovascular peripheral vascular intervention (PVI)**	**Open infrainguinal (INFRA) bypass**	**P-value**
**Data Field**	(n=17,854)	(n=4,474)	
**Total amputations in one year (%)**			<0.001
None	17550 (98.3%)	4409 (98.5%)	
Minor	163 (0.9%)	18 (0.4%)	
BKA	98 (0.5%)	20 (0.4%)	
AKA	43 (0.2%)	27 (0.6%)	
**Major amputations in one year (%)**	141 (0.8%)	47 (1.1%)	0.106
**Days until amputation (mean (SD))**	195.05 (185.9)	434.55 (291.6)	<0.001
**Death in one year (%)**	2265 (54.3%)	6458 (39.0%)	<0.001
**Days of survival (mean (SD))**	1122.52 (894.2)	2113.07 (1582.5)	<0.001
**Primary patency**	NA	2451 (54.8%)	
**Days of patency (mean (SD))**	NA	395.45 (215.83)	
**Readmit within 30 days (%)**	22 (5.1%)	52 (5.9)	0.658
**Days to readmission (mean (SD))**	15.58 (9.71)	16.35 (6.76%)	0.76
**Return to OR within 30 days (%)**	0 (0.0%)	40 (29.9%)	<0.001
**Post-operative MI within 30 days(%)**			<0.001
None	17821 (100.0%)	4434 (99.1%)	
Troponin only	0 (0.0%)	19 (0.4%)	
EKG changes	0 (0.0%)	20 (0.4%)	

© 2025 Bellomo TR et al. *JAMA Network Open.*

eTable 6. Anatomic lesion classification in both open infrainguinal (INFRA) and endovascular peripheral vascular intervention (PVI) data sets

Endovascular peripheral vascular intervention (PVI)	No amputation	Minor amputation below the ankle	Below the knee amputation	Above the knee Amputation	P-value
(n = 17,854)	(n = 17,550)	(n = 163)	(n = 98)	(n = 43)	
**TASCGRADE Lesion 1 (%)**					0.439
A	3352 (19.1%)	27 (16.6%)	16 (16.3%)	3 (7.0%)	
B	4040 (23.0%)	33 (20.2%)	22 (22.4%)	11 (25.6%)	
C	2878 (16.4%)	23 (14.1%)	14 (14.3%)	6 (14.0%)	
D	2299 (13.1%)	27 (16.6%)	10 (10.2%)	10 (23.3%)	
Protect Adjacent Artery	20 (0.1%)	0 (0.0%)	0 (0.0%)	0 (0.0%)	
Missing	4961 (28.3%)	53 (32.5%)	36 (36.7%)	13 (30.2%)	
**TASCGRADE Lesion 2 (%)**					<0.001
A	937 (5.3%)	9 (5.5%)	9 (9.2%)	1 (2.3%)	
B	1092 (6.2%)	16 (9.8%)	8 (8.2%)	1 (2.3%)	
C	822 (4.7%)	9 (5.5%)	9 (9.2%)	4 (9.3%)	
D	782 (4.5%)	18 (11.0%)	6 (6.1%)	5 (11.6%)	
Protect Adjacent Artery	6 (0.0%)	0 (0.0%)	0 (0.0%)	0 (0.0%)	
Missing	13911 (79.3%)	111 (68.1%)	66 (67.3%)	32 (74.4%)	
**TASCGRADE Lesion 4 (%)**					<0.001
A	212 (1.2%)	1 (0.6%)	2 (2.0%)	0 (0.0%)	
B	255 (1.5%)	4 (2.5%)	6 (6.1%)	0 (0.0%)	
C	258 (1.5%)	6 (3.7%)	6 (6.1%)	1 (2.3%)	
D	292 (1.7%)	8 (4.9%)	4 (4.1%)	3 (7.0%)	
Protect Adjacent Artery	1 (0.0%)	0 (0.0%)	0 (0.0%)	0 (0.0%)	
Missing	16532 (94.2%)	144 (88.3%)	80 (81.6%)	39 (90.7%)	

© 2025 Bellomo TR et al. *JAMA Network Open.*

TASCGRADE lesion 5 (%)					<0.001
A	48 (0.3%)	1 (0.6%)	1 (1.0%)	0 (0.0%)	
B	48 (0.3%)	2 (1.2%)	2 (2.0%)	0 (0.0%)	
C	69 (0.4%)	0 (0.0%)	4 (4.1%)	0 (0.0%)	
D	84 (0.5%)	2 (1.2%)	2 (2.0%)	2 (4.7%)	
Missing	17301 (98.6%)	158 (96.9%)	89 (90.8%)	41 (95.3%)	

Open infrainguinal (INFRA) bypass data set	No amputation	Minor amputation below the ankle	Below the knee amputation	Above the knee Amputation	P-value
(n=4,474)	(n = 4,409)	(n = 18)	(n = 20)	(n = 27)	
**GLASS Region Femoropopliteal (%)**					1
Fem-Pop Grade 1	1 (0.0%)	0 (0.0%)	0 (0.0%)	0 (0.0%)	
Fem-Pop Grade 4	1 (0.0%)	0 (0.0%)	0 (0.0%)	0 (0.0%)	
Missing	4407 (100.0%)	18 (100.0%)	20 (100.0%)	27 (100.0%)	
**GLASS Region Infrapopliteal (%)**					1
Infra-Pop Grade 0	1 (0.0%)	0 (0.0%)	0 (0.0%)	0 (0.0%)	
Infra-Pop Grade 1	1 (0.0%)	0 (0.0%)	0 (0.0%)	0 (0.0%)	
Missing	4407 (100.0%)	18 (100.0%)	20 (100.0%)	27 (100.0%)	
**GLASS Region Pedal (%)**					
Missing	4407 (100.0%)	18 (100.0%)	20 (100.0%)	27 (100.0%)	1

© 2025 Bellomo TR et al. *JAMA Network Open*.

eTable 7. Demographics within the open infrainguinal bypass vascular quality initiative data set stratified by graft saphenous vein and prosthetic conduit for initial treatment of claudication

Prosthetic includes Dacron or polytetrafluoroethylene conduits. Abbreviations: GSV, great saphenous vein; BMI, body mass index; PCI, percutaneous coronary intervention; CABG, coronary artery bypass graft; CHF, congestive heart failure; COPD, chronic obstructive pulmonary disease; eGFR, estimated glomerular filtration rate; ASA, American Society of Anesthesiologists; DOAC, Direct oral anticoagulants.

	Great Saphenous Vein Conduit	Prosthetic Conduit	P-value
	(n=2284)	(n=2045)	
Laterality Right (%)	1125 (49.3%)	1014 (49.6%)	0.853
Age (mean (SD))	63.57 (10.54)	65.49 (9.28)	<0.001
Age categories (%)			0.001
<65	1191 (52.1%)	954 (46.7%)	
65-75	817 (35.8%)	790 (38.6%)	
>75	276 (12.1%)	301 (14.7%)	
BMI categories (%)			0.017
<18.5	38 (1.7%)	48 (2.3%)	
18.5-25	518 (22.7%)	539 (26.4%)	
25-30	883 (38.7%)	766 (37.5%)	
30-40	771 (33.8%)	628 (30.7%)	
>40	74 (3.2%)	64 (3.1%)	
Female sex (%)	496 (21.7%)	610 (29.8%)	<0.001
Race (%)			<0.001
White	1993 (87.3%)	1670 (81.7%)	

© 2025 Bellomo TR et al. *JAMA Network Open*.

Black	223 (9.8%)	290 (14.2%)	
Asian	8 (0.4%)	7 (0.3%)	
Other	60 (2.6%)	78 (3.8%)	
Hispanic ethnicity (%)	72 (3.2%)	77 (3.8%)	0.307
Hypertension (%)	1819 (79.6%)	1763 (86.2%)	<0.001
Diabetes (%)			<0.001
None	1593 (69.7%)	1271 (62.2%)	
Diet controlled	98 (4.3%)	74 (3.6%)	
Non-insulin medication	339 (14.8%)	368 (18.0%)	
Insulin dependent	254 (11.1%)	332 (16.2%)	
Smoking (%)			0.012
Never	289 (12.7%)	200 (9.8%)	
Prior	973 (42.6%)	900 (44.0%)	
Current	1022 (44.7%)	945 (46.2%)	
CAD (%)			0.001
None	1752 (76.7%)	1486 (72.7%)	
without MI	146 (6.4%)	122 (6.0%)	
with MI	386 (16.9%)	437 (21.4%)	
CHF (%)	173 (7.6%)	237 (11.6%)	<0.001
Stroke (%)	0 (0.0%)	6 (0.3%)	0.029
COPD (%)	518 (22.7%)	595 (29.1%)	<0.001
Creatinine (mean (SD))	1.04 (0.54%)	1.04 (0.45%)	0.857
eGFR category (%)			0.003
> 60	1797 (78.7%)	1519 (74.3%)	
30-60	423 (18.5%)	455 (22.2%)	
< 30	64 (2.8%)	71 (3.5%)	
ASA Class (%)			<0.001
0	244 (10.7%)	149 (7.3%)	
1	1837 (80.4%)	1583 (77.4%)	

© 2025 Bellomo TR et al. *JAMA Network Open*.

2	203 (8.9%)	313 (15.3%)	
Statin (%)			0.012
No	608 (26.6%)	465 (22.7%)	
Yes	1619 (70.9%)	1533 (75.0%)	
No for medical reasons	41 (1.8%)	39 (1.9%)	
Non-compliant	16 (0.7%)	8 (0.4%)	
Aspirin (%)			0.003
No	571 (25.0%)	447 (21.9%)	
Yes	1647 (72.1%)	1510 (73.8%)	
No for medical reasons	55 (2.4%)	63 (3.1%)	
Non-compliant	11 (0.5%)	25 (1.2%)	
Clopidogrel (%)	384 (16.8%)	472 (23.1%)	<0.001
Prasugrel (%)	11 (0.5%)	13 (0.6%)	0.634
Ticagrelor (%)	9 (0.4%)	8 (0.4%)	1
DOAC (%)	154 (6.7%)	150 (7.3%)	0.483
Warfarin (%)	112 (4.9%)	106 (5.2%)	0.726
Length of stay (mean (SD))	3.82 (8.10)	4.33 (18.37)	0.226

© 2025 Bellomo TR et al. *JAMA Network Open*.

eFigure 1. Flowchart of inclusion and exclusion of index endovascular and open procedures for comparative analysis

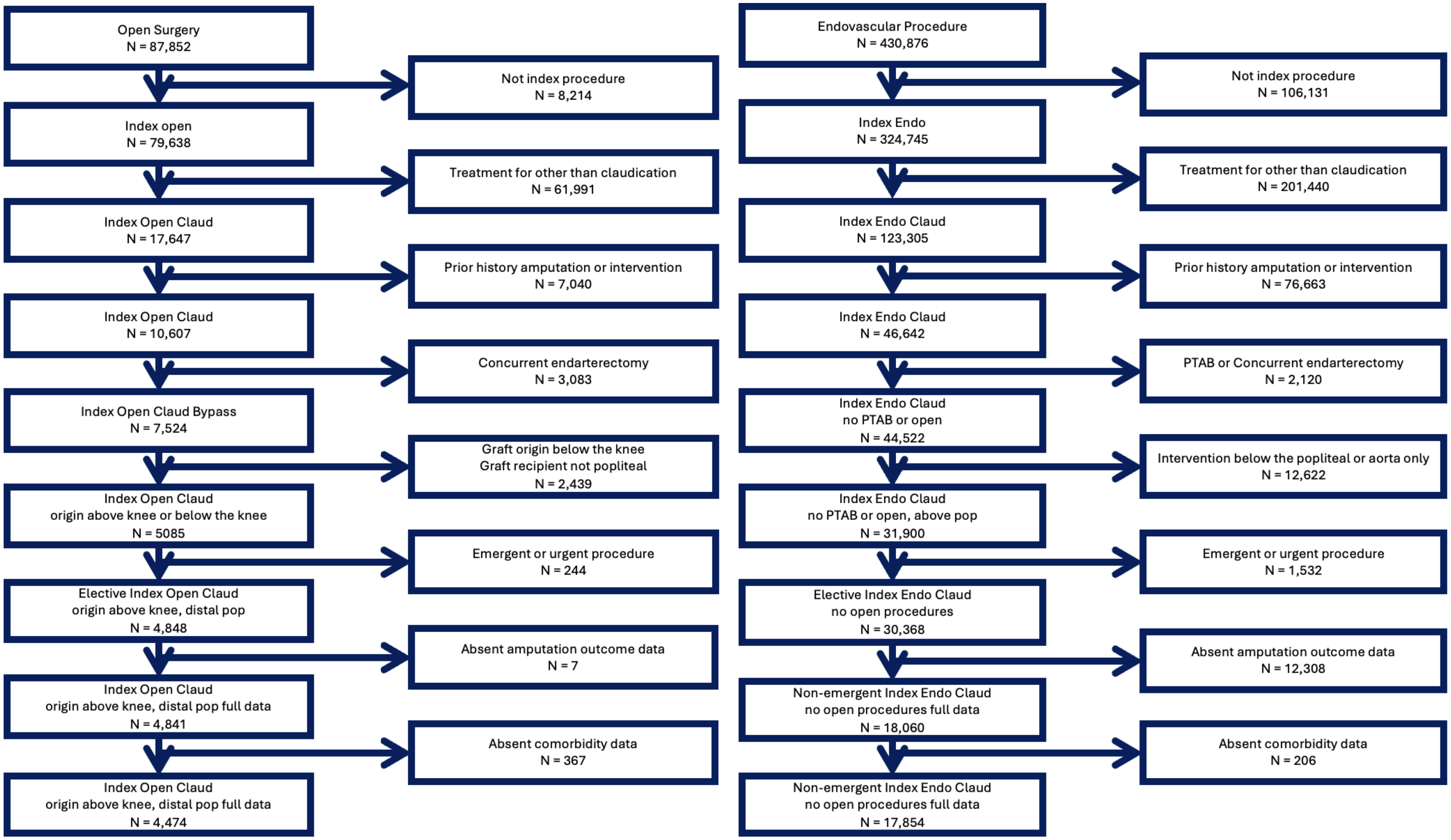

Abbreviations: PTAB, Percutaneous Transmural Arterial Bypass.

© 2025 Bellomo TR et al. *JAMA Network Open*.

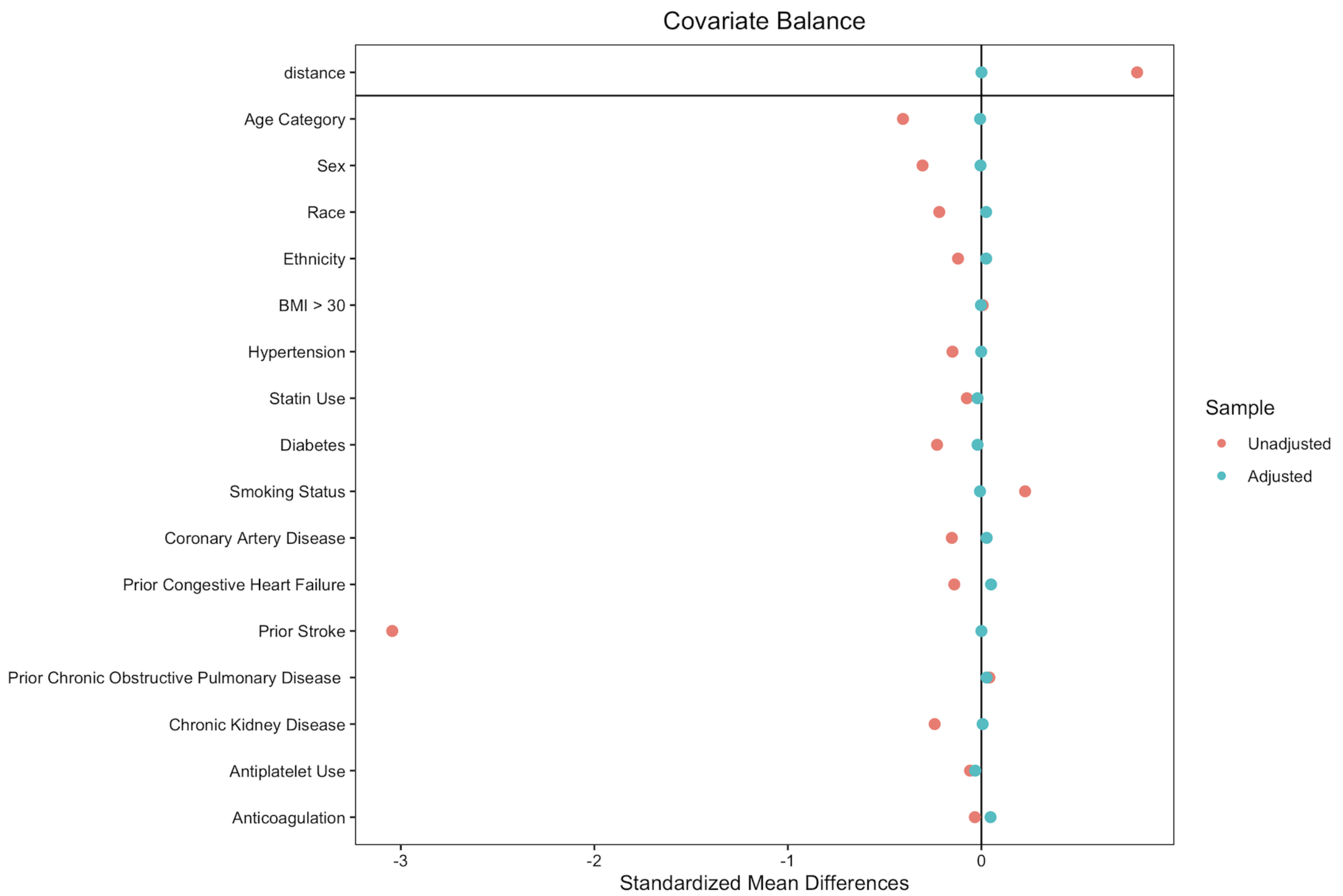

eFigure 2. Love plot illustrating standardized mean differences in patient comorbidities before and after propensity score matching of patients undergoing endovascular procedures to 4,474 patients undergoing open procedures for claudication.

© 2025 Bellomo TR et al. *JAMA Network Open*.

A)

One Year Major Amputation	Group	Status	Affected/Total (%)	Incidence (1000 p-y)	p-value	p-interaction	HR (95% CI)
Demographics	Total		77 / 8948 (0.9 %)	3.47	0.007		0.53 (0.33 to 0.84)
	Age	Age<65	51 / 4450 (1.1 %)	4.42	0.005	0.444	0.43 (0.24 to 0.77)
		Age 65-75	21 / 3293 (0.6 %)	2.64	0.461		0.72 (0.30 to 1.72)
		Age>75	5 / 1205 (0.4 %)	1.85	0.454		0.50 (0.08 to 3.06)
	Sex	Male	53 / 6659 (0.8 %)	3.17	0.007	0.305	0.46 (0.26 to 0.81)
		Female	24 / 2289 (1 %)	4.38	0.223		0.60 (0.26 to 1.36)
	Race	White	61 / 7622 (0.8 %)	3.21	0.004	0.249	0.46 (0.27 to 0.78)
		Black	14 / 1020 (1.4 %)	5.45	0.233		0.51 (0.17 to 1.54)
		Other	2 / 306 (0.7 %)	3.21	0.999		557581291.29 (0.00 to Inf)
Comorbidities	Diabetes	Yes	35 / 3054 (1.1 %)	4.76	0.901	0.003	0.96 (0.49 to 1.89)
		No	42 / 5894 (0.7 %)	2.83	<0.0001		0.27 (0.13 to 0.55)
	Statin	Yes	52 / 6528 (0.8 %)	3.29	0.001	0.099	0.36 (0.20 to 0.65)
		No	25 / 2420 (1 %)	3.91	0.902		0.95 (0.43 to 2.09)
	Antiplatlet	Yes	58 / 6962 (0.8 %)	3.36	0.017	0.959	0.52 (0.31 to 0.89)
		No	19 / 1986 (1 %)	3.83	0.097		0.45 (0.17 to 1.16)
	Anticoagulant	Yes	14 / 1021 (1.4 %)	6.70	0.72	0.622	0.82 (0.29 to 2.38)
		No	63 / 7927 (0.8 %)	3.13	0.003		0.46 (0.27 to 0.76)
	Smoking	Current	40 / 4092 (1 %)	3.77	0.004		0.38 (0.19 to 0.73)
		Prior	32 / 3816 (0.8 %)	3.53	0.046		0.48 (0.23 to 0.99)
		Never	5 / 1040 (0.5 %)	1.96	0.999		447560906.84 (0.00 to Inf)

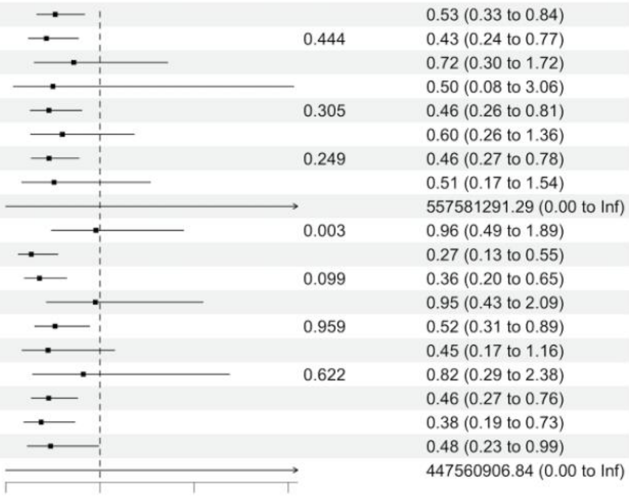

B)

One Year Death	Group	Status	Affected/Total (%)	Incidence (1000 p-y)	p-value	p-interaction	HR (95% CI)
Demographics	Total		3777 / 8948 (42.2 %)	94.14	<0.0001		1.67 (1.54 to 1.80)
	Age	Age<65	1875 / 4450 (42.1 %)	92.44	<0.0001	0.973	1.62 (1.45 to 1.80)
		Age 65-75	1415 / 3293 (43 %)	96.54	<0.0001		1.71 (1.51 to 1.93)
		Age>75	487 / 1205 (40.4 %)	94.04	<0.0001		1.64 (1.33 to 2.02)
	Sex	Male	2832 / 6659 (42.5 %)	94.64	<0.0001	0.639	1.67 (1.53 to 1.82)
		Female	945 / 2289 (41.3 %)	92.69	<0.0001		1.58 (1.36 to 1.84)
	Race	White	3214 / 7622 (42.2 %)	93.34	<0.0001	0.574	1.66 (1.53 to 1.80)
		Black	443 / 1020 (43.4 %)	99.13	<0.0001		1.63 (1.31 to 2.03)
		Other	120 / 306 (39.2 %)	98.61	0.056		1.53 (0.99 to 2.36)
Comorbidities	Diabetes	Yes	1249 / 3054 (40.9 %)	94.41	<0.0001	0.529	1.56 (1.37 to 1.78)
		No	2528 / 5894 (42.9 %)	94.01	<0.0001		1.70 (1.55 to 1.87)
	Statin	Yes	2767 / 6528 (42.4 %)	96.33	<0.0001	0.108	1.60 (1.47 to 1.75)
		No	1010 / 2420 (41.7 %)	88.64	<0.0001		1.80 (1.55 to 2.08)
	Antiplatlet	Yes	2927 / 6962 (42 %)	94.46	<0.0001	0.145	1.61 (1.47 to 1.75)
		No	850 / 1986 (42.8 %)	93.07	<0.0001		1.81 (1.54 to 2.14)
	Anticoagulant	Yes	437 / 1021 (42.8 %)	118.57	0.013	0.031	1.31 (1.06 to 1.62)
		No	3340 / 7927 (42.1 %)	91.67	<0.0001		1.72 (1.58 to 1.86)
	Smoking	Current	1748 / 4092 (42.7 %)	92.22	<0.0001	0.494	1.67 (1.49 to 1.87)
		Prior	1620 / 3816 (42.5 %)	97.21	<0.0001		1.60 (1.42 to 1.79)
		Never	409 / 1040 (39.3 %)	90.91	<0.0001		1.80 (1.43 to 2.26)

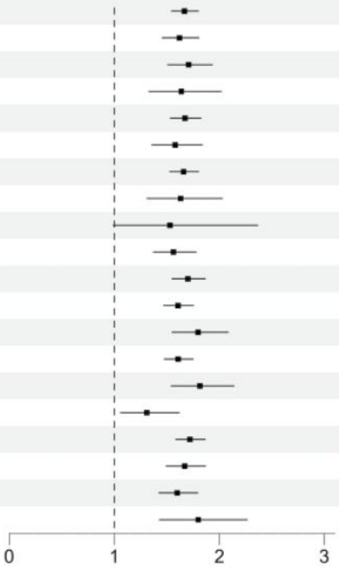

© 2025 Bellomo TR et al. *JAMA Network Open*.

eFigure 3. Risk of adverse outcomes in propensity matched endovascular compared to open intervention by clinically relevant subgroups

A) Outcomes of one-year major amputation above the ankle and B) death within one year used the reference category intervention of index open infrainguinal procedures. Hazard ratios with corresponding 95% confidence intervals were reported based on Cox proportional hazards regression models with covariates of age, sex, self-reported race, ethnicity, obesity, smoking status, hypertension, diabetes, coronary artery disease, congestive heart failure, stroke, chronic obstructive pulmonary disease, chronic kidney disease, statin, antiplatelet, and anticoagulation. Subgroup analyses were performed by splitting cohorts based on presence or absence of relevant subgroups. An interaction p value was calculated to identify significant interactions between subgroups and index procedure type influencing amputation risk.

© 2025 Bellomo TR et al. *JAMA Network Open*.

One Year Death	Group	Status	Affected/Total (%)	Incidence (1000 p-y)	p-value	p-interaction	HR (95% CI)
Demographics	Total		8723 / 22328 (39.1 %)	107.95	<0.0001		2.09 (1.97 to 2.23)
	Age	Age<65	3313 / 8256 (40.1 %)	102.49	<0.0001	0.908	2.03 (1.86 to 2.22)
		Age 65-75	3511 / 8865 (39.6 %)	109.96	<0.0001		2.23 (2.02 to 2.46)
		Age>75	1899 / 5207 (36.5 %)	114.73	<0.0001		2.17 (1.86 to 2.53)
	Sex	Male	5683 / 14272 (39.8 %)	106.78	<0.0001	0.943	2.11 (1.97 to 2.27)
		Female	3040 / 8056 (37.7 %)	110.20	<0.0001		2.28 (2.03 to 2.56)
	Race	White	6836 / 17547 (39 %)	105.72	<0.0001	0.995	2.12 (1.98 to 2.26)
		Black	1414 / 3517 (40.2 %)	111.55	<0.0001		2.24 (1.89 to 2.66)
		Other	473 / 1264 (37.4 %)	136.34	<0.0001		2.40 (1.77 to 3.25)
Comorbidities	Diabetes	Yes	3634 / 9450 (38.5 %)	112.06	<0.0001	0.714	2.16 (1.95 to 2.39)
		No	5089 / 12878 (39.5 %)	105.19	<0.0001		2.14 (1.98 to 2.31)
	Statin	Yes	6620 / 16789 (39.4 %)	110.84	<0.0001	0.324	2.11 (1.97 to 2.26)
		No	2103 / 5539 (38 %)	99.75	<0.0001		2.29 (2.03 to 2.59)
	Antiplatlet	Yes	6870 / 17663 (38.9 %)	107.03	<0.0001	0.003	2.07 (1.94 to 2.22)
		No	1853 / 4665 (39.7 %)	111.49	<0.0001		2.48 (2.18 to 2.84)
	Anticoagulant	Yes	1177 / 2918 (40.3 %)	132.15	<0.0001	0.002	1.71 (1.46 to 2.01)
		No	7546 / 19410 (38.9 %)	104.95	<0.0001		2.23 (2.09 to 2.38)
	Smoking	Current	3462 / 8580 (40.3 %)	104.79	<0.0001	0.11	2.19 (2.00 to 2.41)
		Prior	3852 / 10015 (38.5 %)	108.05	<0.0001		2.01 (1.84 to 2.20)
		Never	1409 / 3733 (37.7 %)	116.25	<0.0001		2.53 (2.11 to 3.02)

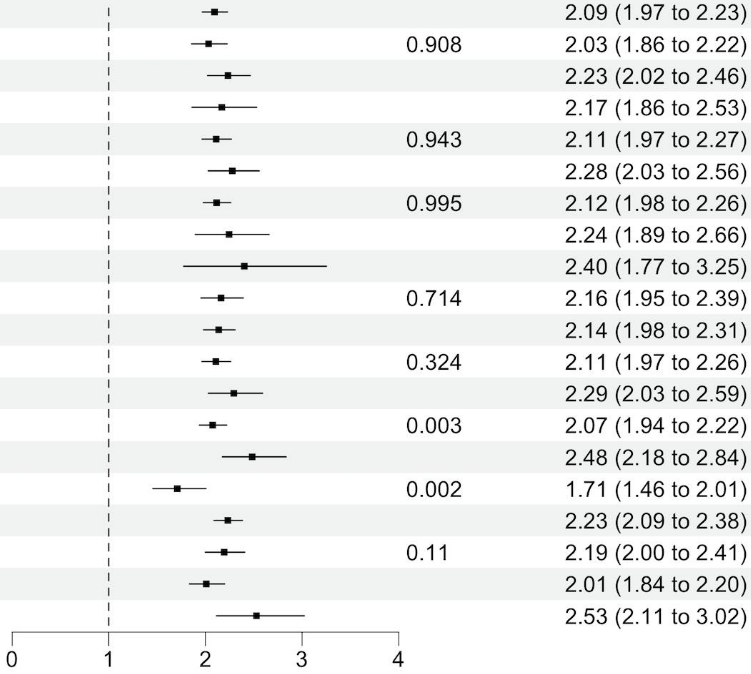

eFigure 4. Risk of major amputation and death using endovascular compared to open intervention by demographic subgroups
The reference category intervention was index open infrainguinal procedures. Hazard ratios with corresponding 95% confidence intervals were reported based on Cox proportional hazards regression models with covariates of age, sex, and self-reported race, ethnicity, obesity, smoking status, hypertension, diabetes, coronary artery disease, congestive heart failure, stroke, chronic obstructive pulmonary disease, chronic kidney disease, statin medication, antiplatelet, and anticoagulation. Subgroup analyses were performed by splitting cohorts based on presence or absence of demographic. An interaction p value was calculated to identify significant interactions between demographic subgroups and index procedure type influencing amputation risk.

© 2025 Bellomo TR et al. *JAMA Network Open*.

30 Day Readmission	Group	Status	Affected/Total (%)	Incidence (1000 p-y)	p-value	p-interaction	HR (95% CI)
30 Day Readmission	Total		74 / 22328 (0.3 %)	20921.77	0.008		0.24 (0.08 to 0.68)
	Age	Age<65	38 / 8256 (0.5 %)	19985.59	0.149	0.263	0.47 (0.17 to 1.31)
		Age 65-75	24 / 8865 (0.3 %)	24676.06	0.267		0.44 (0.11 to 1.86)
		Age>75	12 / 5207 (0.2 %)	18099.17	0.485		0.60 (0.14 to 2.54)
	Sex	Male	39 / 14272 (0.3 %)	21309.88	0.222	0.585	0.51 (0.17 to 1.50)
		Female	35 / 8056 (0.4 %)	20505.62	0.197		0.53 (0.20 to 1.39)
	Race	White	60 / 17547 (0.3 %)	21769.38	0.051	0.946	0.43 (0.18 to 1.00)
		Black	11 / 3517 (0.3 %)	17380.95	0.338		0.49 (0.12 to 2.10)
		Other	3 / 1264 (0.2 %)	20277.78	NA		

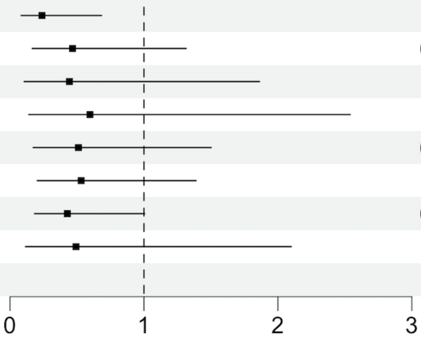

eFigure 5. Risk of readmission and myocardial infarction using endovascular compared to open intervention by demographic subgroups
The reference category intervention was index open infrainguinal procedures. Hazard ratios with corresponding 95% confidence intervals were reported based on Cox proportional hazards regression models with covariates of age, sex, and self-reported race, ethnicity, obesity, smoking status, hypertension, diabetes, coronary artery disease, congestive heart failure, stroke, chronic obstructive pulmonary disease, chronic kidney disease, statin medication, antiplatelet, and anticoagulation. Subgroup analyses were performed by splitting cohorts based on presence or absence of demographic. An interaction p value was calculated to identify significant interactions between demographic subgroups and index procedure type influencing adverse event risk.

© 2025 Bellomo TR et al. *JAMA Network Open*.

One Year Primary Patency	Group	Status	Affected/Total (%)	Incidence (1000 p-y)	p-value	p-interaction	HR (95% CI)
Demographics	Total		4238 / 4474 (94.7 %)	313.46	0.234		0.96 (0.90 to 1.02)
	Age	Age<65	2089 / 2226 (93.8 %)	296.47	0.062	0.045	0.92 (0.84 to 1.00)
		Age 65-75	1584 / 1655 (95.7 %)	332.80	0.982		1.00 (0.91 to 1.11)
		Age>75	565 / 593 (95.3 %)	329.65	0.144		1.13 (0.96 to 1.34)
	Sex	Male	3163 / 3334 (94.9 %)	316.65	0.163	0.128	0.95 (0.88 to 1.02)
		Female	1075 / 1140 (94.3 %)	304.45	0.343		1.06 (0.94 to 1.20)
	Race	White	3591 / 3792 (94.7 %)	312.33	0.748	0.191	0.99 (0.93 to 1.06)
		Black	496 / 522 (95 %)	318.64	0.441		0.93 (0.78 to 1.12)
		Other	151 / 160 (94.4 %)	324.26	0.258		0.83 (0.59 to 1.15)
Comorbidities	Diabetes	Yes	1417 / 1506 (94.1 %)	314.24	0.914	0.612	0.99 (0.89 to 1.11)
		No	2821 / 2968 (95 %)	313.08	0.421		0.97 (0.90 to 1.05)
	Statin	Yes	3063 / 3244 (94.4 %)	318.86	0.696	0.524	0.99 (0.92 to 1.06)
		No	1175 / 1230 (95.5 %)	300.23	0.355		0.95 (0.84 to 1.06)
	Antiplatlet	Yes	3269 / 3451 (94.7 %)	319.51	0.468	0.972	0.97 (0.91 to 1.04)
		No	969 / 1023 (94.7 %)	294.65	0.84		0.99 (0.87 to 1.12)
	Anticoagulant	Yes	524 / 545 (96.1 %)	399.45	0.409	0.348	1.08 (0.90 to 1.28)
		No	3714 / 3929 (94.5 %)	304.22	0.304		0.97 (0.90 to 1.03)
	Smoking	Current	1914 / 2028 (94.4 %)	303.94	0.341	0.721	0.96 (0.87 to 1.05)
		Prior	1829 / 1933 (94.6 %)	319.00	0.534		1.03 (0.94 to 1.13)
		Never	495 / 513 (96.5 %)	332.43	0.205		0.89 (0.74 to 1.07)

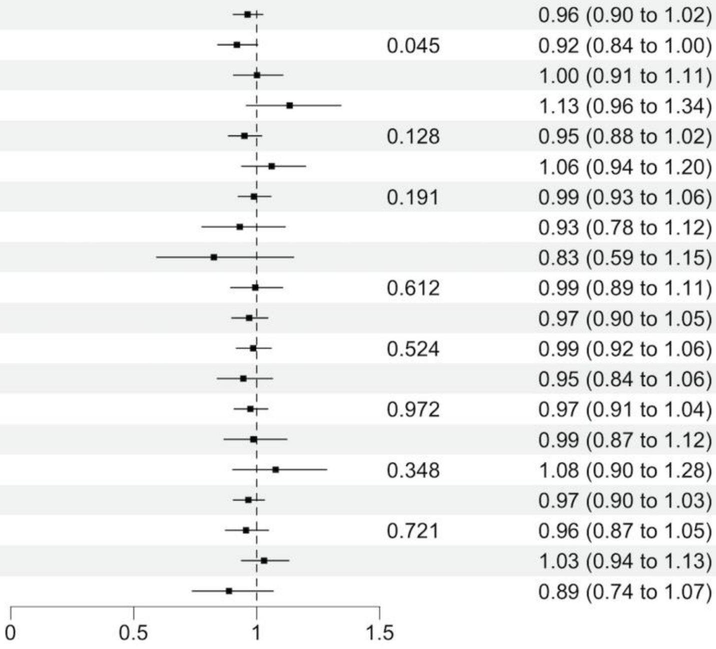

eFigure 6. Risk of major amputation and primary patency after one year using prosthetic conduit compared to great saphenous vein for initial treatment of claudication by clinically relevant subgroups

The reference category conduit was great saphenous vein. Hazard ratios with corresponding 95% confidence intervals were reported based on Cox proportional hazards regression models with covariates of age, sex, and self-reported race, ethnicity, obesity, smoking status, hypertension, diabetes, coronary artery disease, congestive heart failure, stroke, chronic obstructive pulmonary disease, chronic kidney disease, statin medication, antiplatelet, anticoagulation, and graft recipient. Subgroup analyses were performed by splitting cohorts based on presence or absence of the clinical subgroup. An interaction p value was calculated to identify significant interactions between subgroups and intervention category influencing amputation and primary patency risk.

© 2025 Bellomo TR et al. *JAMA Network Open*.

30 Day Outcomes	Group	Status	Affected/Total (%)	Incidence (1000 p-y)	p-value	p-interaction	HR (95% CI)
30 Day Return to OR	Total		449 / 4474 (10 %)	18.60	<0.0001		0.63 (0.52 to 0.77)
	Age	Age<65	261 / 2226 (11.7 %)	21.85	0.001	0.978	0.65 (0.50 to 0.84)
		Age 65-75	138 / 1655 (8.3 %)	15.31	0.04		0.70 (0.49 to 0.98)
		Age>75	50 / 593 (8.4 %)	15.71	0.047		0.55 (0.30 to 0.99)
	Sex	Male	330 / 3334 (9.9 %)	18.37	0.001	0.489	0.67 (0.53 to 0.85)
		Female	119 / 1140 (10.4 %)	19.26	0.003		0.57 (0.39 to 0.83)
	Race	White	380 / 3792 (10 %)	18.39	<0.0001	0.512	0.67 (0.54 to 0.83)
		Black	55 / 522 (10.5 %)	20.70	0.024		0.53 (0.31 to 0.92)
		Other	14 / 160 (8.8 %)	16.99	0.407		0.64 (0.22 to 1.85)
30 Day Readmission	Total		52 / 4474 (1.2 %)	2.01	0.553		0.84 (0.48 to 1.48)
	Age	Age<65	28 / 2226 (1.3 %)	2.16	0.438	0.683	1.34 (0.64 to 2.81)
		Age 65-75	17 / 1655 (1 %)	1.78	0.164		0.48 (0.17 to 1.35)
		Age>75	7 / 593 (1.2 %)	2.08	0.627		1.45 (0.32 to 6.55)
	Sex	Male	29 / 3334 (0.9 %)	1.51	0.612	0.683	0.82 (0.39 to 1.74)
		Female	23 / 1140 (2 %)	3.45	0.874		1.07 (0.47 to 2.44)
	Race	White	47 / 3792 (1.2 %)	2.12	0.827	0.67	1.07 (0.60 to 1.90)
		Black	5 / 522 (1 %)	1.75	0.475		0.52 (0.09 to 3.13)
		Other	0 / 160 (0 %)	0.00	NA		

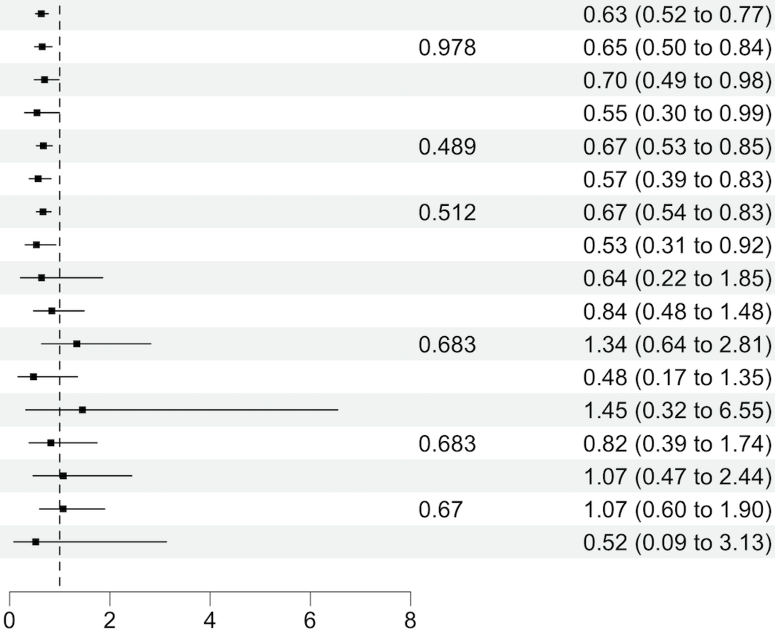

eFigure 7. Risk of outcomes using prosthetic conduit compared to great saphenous vein for initial treatment of claudication stratified by demographic subgroups

The reference category for conduit was great saphenous vein. Hazard ratios with corresponding 95% confidence intervals were reported based on Cox proportional hazards regression models with covariates of age, sex, and self-reported race, ethnicity, obesity, smoking status, hypertension, diabetes, coronary artery disease, congestive heart failure, stroke, chronic obstructive pulmonary disease, chronic kidney disease, statin medication, antiplatelet, anticoagulation, and graft recipient. Subgroup analyses were performed by splitting cohorts based on demographic group. An interaction p value was calculated to identify significant interactions between demographic subgroups and intervention category influencing amputation or patency risk.

© 2025 Bellomo TR et al. *JAMA Network Open*.

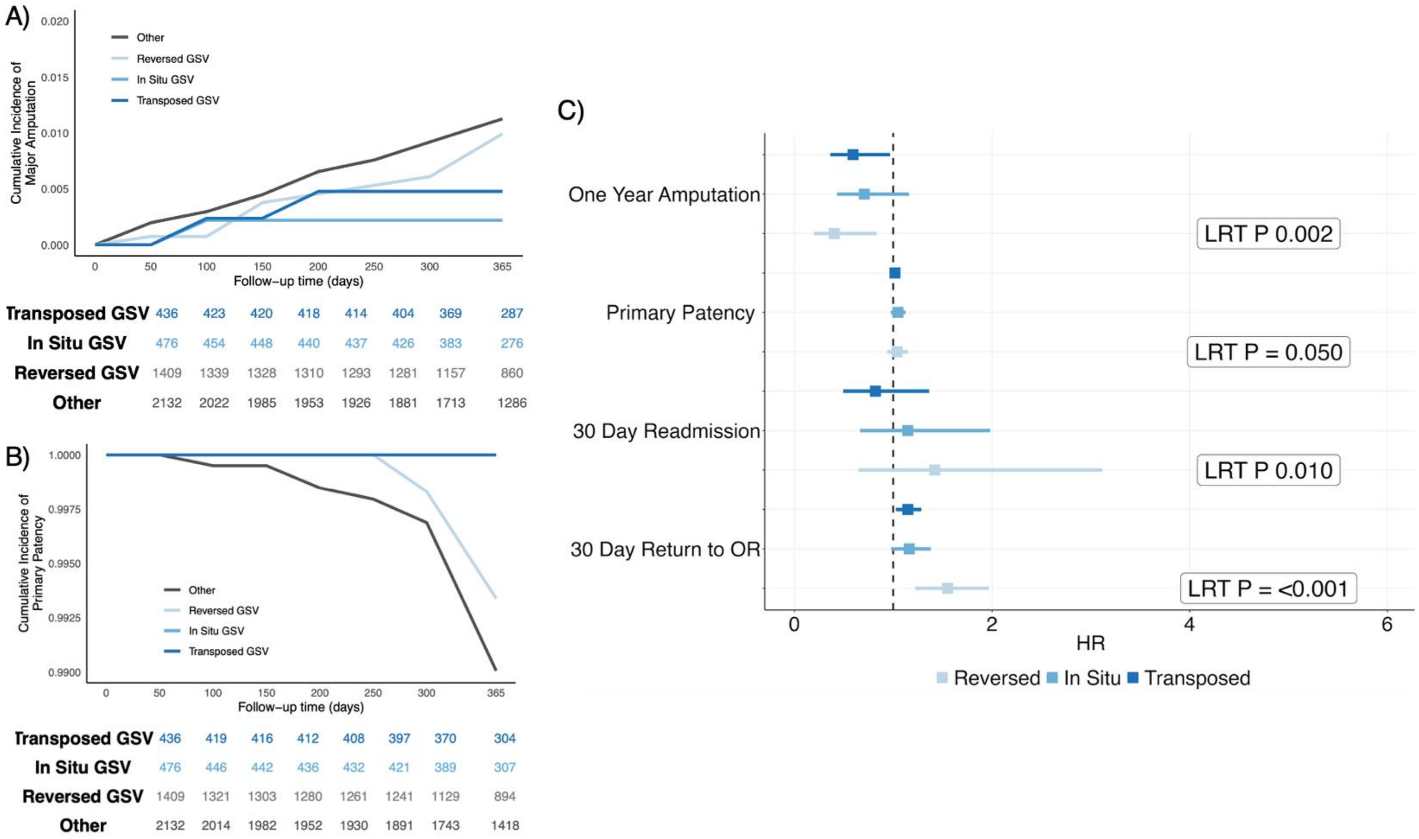

eFigure 8. Risk of adverse outcomes stratified by GSV configuration type for initial treatment of claudication
Kaplan Meier curve of (A) major amputation and (B) primary patency over one year. (C) Hazard ratios with corresponding 95% confidence intervals were reported based on Cox proportional hazards regression models with covariates of age, sex, and self-reported race, ethnicity, obesity, smoking status, hypertension, diabetes, coronary artery disease, congestive heart failure, stroke, chronic obstructive pulmonary disease, chronic kidney disease, statin medication, antiplatelet, anticoagulant, and graft recipient. The reference category for each group was the GSV configuration compared to the category of all other non-GSV conduits. Likelihood ratio test was used to compare differences in goodness of fit between the three GSV configuration models. Light blue represents reversed great

© 2025 Bellomo TR et al. *JAMA Network Open*.

saphenous vein, medium blue represents in situ great saphenous vein, dark blue represents transposed great saphenous vein, and grey represents all other conduits.

© 2025 Bellomo TR et al. *JAMA Network Open*.

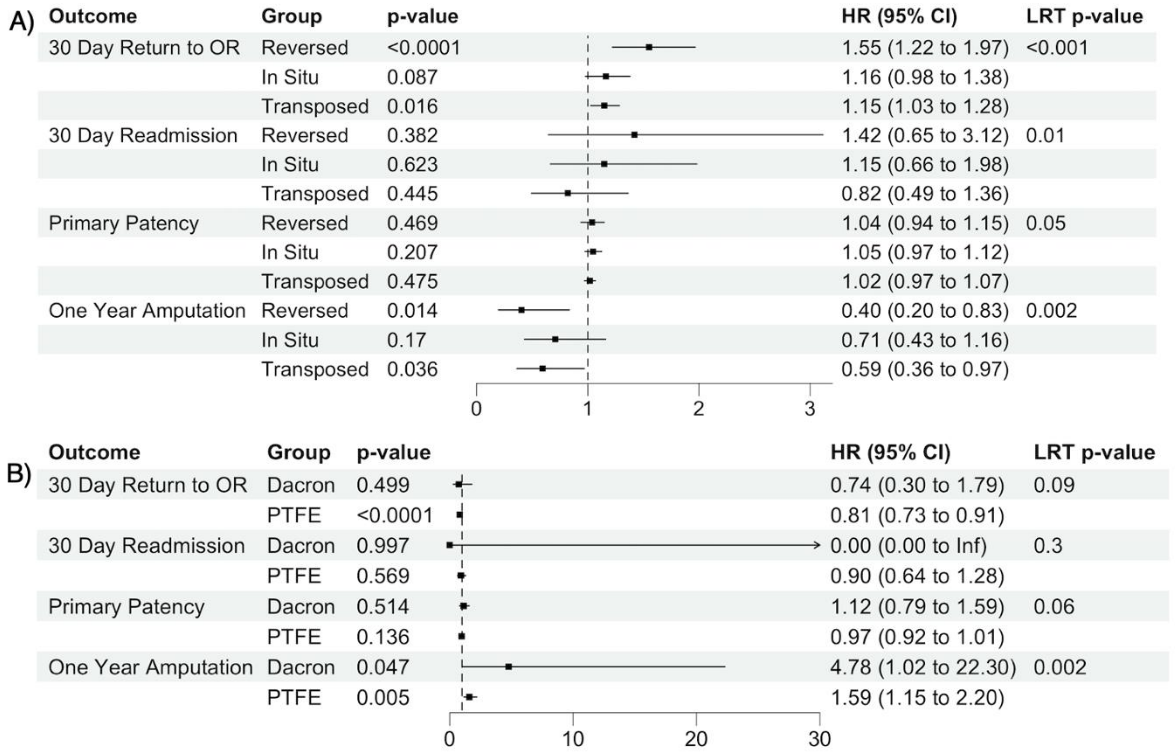

eFigure 9. Risk of adverse outcomes stratified A) great saphenous vein and B) prosthetic conduit type for initial treatment of claudication by demographic subgroups

The reference category for each group was the great saphenous vein conduit compared to the category of other. Hazard ratios with corresponding 95% confidence intervals were reported based on Cox proportional hazards regression models with covariates of age, sex, and self-reported race, ethnicity, obesity, smoking status, hypertension, diabetes, coronary artery disease, congestive heart failure, stroke, chronic obstructive pulmonary disease, chronic kidney disease, statin medication, antiplatelet, anticoagulation, and graft recipient. Likelihood ratio test was used to compare differences in goodness of fit between all three great saphenous vein specific models.

© 2025 Bellomo TR et al. *JAMA Network Open*.

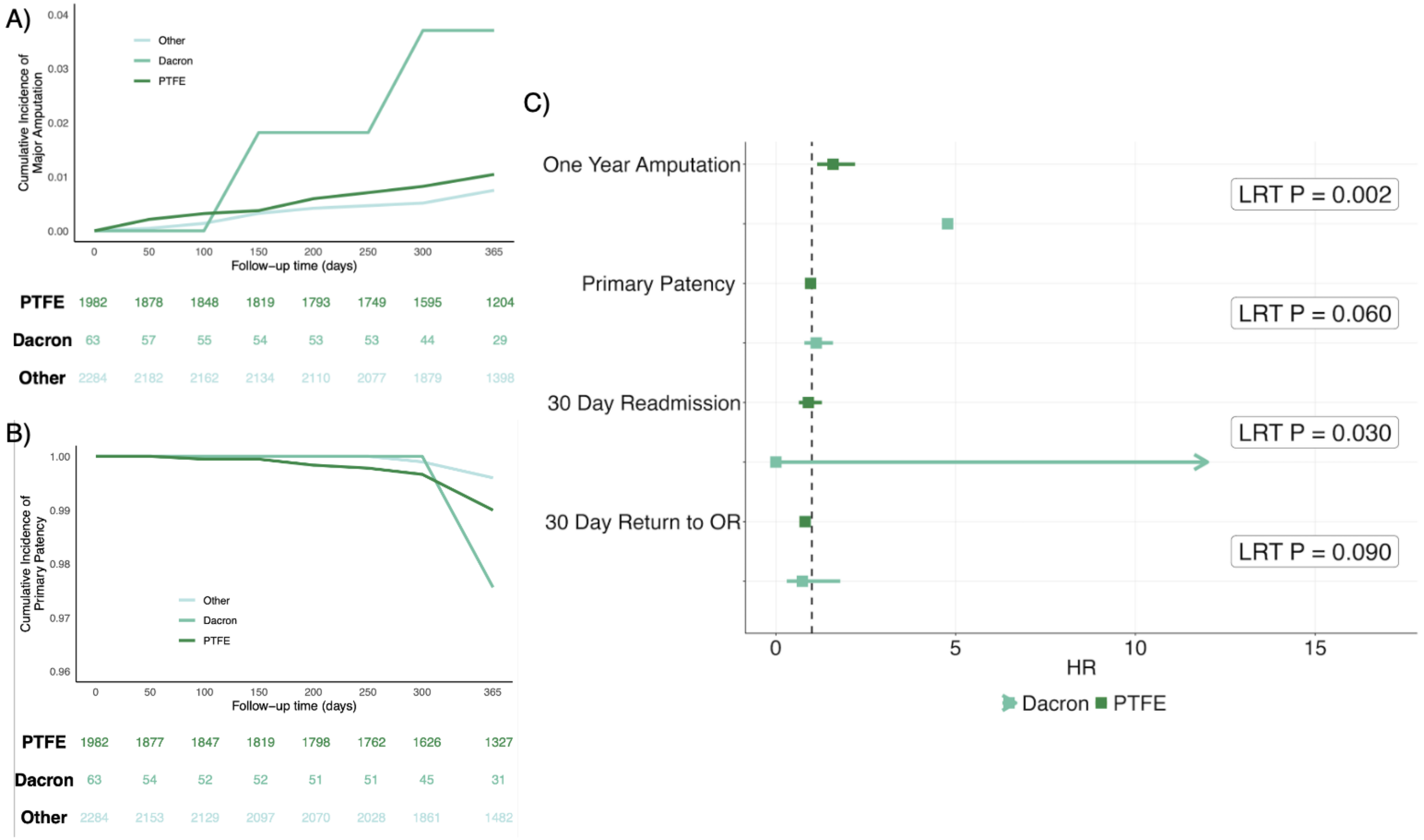

eFigure 10. Risk of adverse outcomes stratified by prosthetic conduit type for initial treatment of claudication
Kaplan Meier curve of (A) primary patency and (B) major amputation over one year. (C) Hazard ratios with corresponding 95% confidence intervals were reported based on Cox proportional hazards regression models with covariates of age, sex, and self-reported race, ethnicity, obesity, smoking status, hypertension, diabetes, coronary artery disease, congestive heart failure, stroke, chronic obstructive pulmonary disease, chronic kidney disease, statin medication, antiplatelet, anticoagulant, and graft recipient. The reference category for each group was the prosthetic conduit compared to the category of all other non-prosthetic conduits. Likelihood ratio test was used to compare differences in goodness of fit between two prosthetic conduit specific models. Dark green represents Polytetrafluoroethylene (PTFE), medium green represents dacron, and light green represents other conduit.

© 2025 Bellomo TR et al. *JAMA Network Open*.

eReferences.

1. Conte MS, Pomposelli FB, Clair DG, Geraghty PJ, McKinsey JF, Mills JL, Moneta GL, Murad MH, Powell RJ, Reed AB, Schanzer A, Sidawy AN. Society for Vascular Surgery practice guidelines for atherosclerotic occlusive disease of the lower extremities: Management of asymptomatic disease and claudication. *Journal of Vascular Surgery*. 2015;61(3):2S-41S.e1.
2. Bradbury AW, Adam DJ, Bell J, Forbes JF, Fowkes FGR, Gillespie I, Ruckley CV, Raab GM. Bypass versus Angioplasty in Severe Ischaemia of the Leg (BASIL) trial: An intention-to-treat analysis of amputation-free and overall survival in patients randomized to a bypass surgery-first or a balloon angioplasty-first revascularization strategy. *Journal of Vascular Surgery*. 2010;51(5 SUPPL.):5S-17S.

© 2025 Bellomo TR et al. *JAMA Network Open*.
