# Supplementary material for: Endovascular Therapy, Open Surgical Bypass, and Conduit Types for Index Treatment of Claudication
Source: JAMA Netw Open. 2025 Oct 16;8(10):e2533352. doi: 10.1001/jamanetworkopen.2025.33352 (PMC12531885; doi:10.1001/jamanetworkopen.2025.33352)
Supplement: Supplement 3. — Data Sharing Statement [file jamanetwopen-e2533352-s003.pdf]

Data Sharing Statement

Bellomo. Endovascular Therapy, Open Surgical Bypass, and Conduit Types for Index Treatment of Claudication. *JAMA Netw Open*. Published September 23, 2025. doi:10.1001/jamanetworkopen.2025.33352

Data
Data available: Yes
Data types: Deidentified participant data
How to access data: These data are available for request from the VQI.
When available: With publication

Supporting Documents
Document types: None

Additional Information
Who can access the data: Data will be made available to researchers on reasonable request
Types of analyses: Data will be made available to researchers on reasonable request
Mechanisms of data availability: Data will be made available to researchers on reasonable request upon application to the VQI registry.
